# Principal and independent genomic components of brain structure and function

**DOI:** 10.1111/gbb.12876

**Published:** 2024-01-15

**Authors:** Lennart M. Oblong, Sourena Soheili‐Nezhad, Nicolò Trevisan, Yingjie Shi, Christian F. Beckmann, Emma Sprooten

**Affiliations:** ^1^ Department of Cognitive Neuroscience Donders Institute for Brain, Cognition and Behaviour, Radboud University Medical Centre Nijmegen The Netherlands; ^2^ Language and Genetics Department Max Planck Institute for Psycholinguistics Nijmegen The Netherlands; ^3^ Department of Human Genetics Donders Institute for Brain, Cognition and Behaviour, Radboud University Medical Centre Nijmegen The Netherlands; ^4^ Centre for Cognitive Neuroimaging Donders Institute for Brain, Cognition and Behaviour, Radboud University Nijmegen The Netherlands

**Keywords:** genomic ICA, genomic PCA, genomics, GWAS, MELODIC, MRI, neuroimaging genetics, quantitative genetics, statistical genetics

## Abstract

The highly polygenic and pleiotropic nature of behavioural traits, psychiatric disorders and structural and functional brain phenotypes complicate mechanistic interpretation of related genome‐wide association study (GWAS) signals, thereby obscuring underlying causal biological processes. We propose genomic principal and independent component analysis (PCA, ICA) to decompose a large set of univariate GWAS statistics of multimodal brain traits into more interpretable latent genomic components. Here we introduce and evaluate this novel methods various analytic parameters and reproducibility across independent samples. Two UK Biobank GWAS summary statistic releases of 2240 imaging‐derived phenotypes (IDPs) were retrieved. Genome‐wide beta‐values and their corresponding standard‐error scaled *z*‐values were decomposed using genomic PCA/ICA. We evaluated variance explained at multiple dimensions up to 200. We tested the inter‐sample reproducibility of output of dimensions 5, 10, 25 and 50. Reproducibility statistics of the respective univariate GWAS served as benchmarks. Reproducibility of 10‐dimensional PCs and ICs showed the best trade‐off between model complexity and robustness and variance explained (PCs: |*r*_*z*_ − max| = 0.33, |*r*_raw_ − max| = 0.30; ICs: |*r*_*z*_ − max| = 0.23, |*r*_raw_ − max| = 0.19). Genomic PC and IC reproducibility improved substantially relative to mean univariate GWAS reproducibility up to dimension 10. Genomic components clustered along neuroimaging modalities. Our results indicate that genomic PCA and ICA decompose genetic effects on IDPs from GWAS statistics with high reproducibility by taking advantage of the inherent pleiotropic patterns. These findings encourage further applications of genomic PCA and ICA as fully data‐driven methods to effectively reduce the dimensionality, enhance the signal to noise ratio and improve interpretability of high‐dimensional multitrait genome‐wide analyses.

## Graphical abstract

We propose genomic principal and independent component analysis (PCA, ICA) to decompose a large set of univariate genome‐wide association study (GWAS) statistics of multimodal brain traits into more interpretable latent genomic components. Our results indicate that genomic PCA and ICA decompose genetic effects on imaging derived phenotypes from GWAS statistics with high reproducibility by taking advantage of the inherent pleiotropic patterns. Genomic components clustered along the boundaries of neuroimaging modalities. These findings encourage further applications of genomic PCA and ICA as fully data‐driven methods to effectively reduce the dimensionality, enhance the signal to noise ratio and improve interpretability of high‐dimensional multitrait genome‐wide analyses.

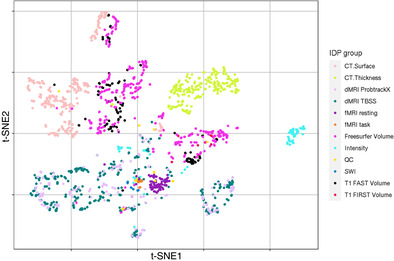

## 1 INTRODUCTION

Individual differences in brain structure and function are determined by complex biological mechanisms that remain largely unknown. Measures of human brain structure and function, as assessed through magnetic resonance imaging (MRI), are heritable.^1^, ^2^, ^3^, ^4^, ^5^, ^6^, ^7^, ^8^, ^9^, ^10^ Genome‐wide association studies (GWAS) have emerged as a popular and powerful tool for estimating the effects of common genetic variants on behavioural traits, psychiatric disorders and structural and functional brain phenotypes. GWAS output consists of mass‐univariate statistics of millions of single nucleotide polymorphisms (SNPs) quantifying the typically small, genome‐wide associations of common SNPs with a trait of interest. The highly polygenic and pleiotropic nature of brain features and multifactorial behavioural and psychiatric phenotypes,^1^, ^8^, ^11^, ^12^ complicate the interpretation of GWAS in terms of clear underlying causal biological processes.^13^, ^14^, ^15^ SNPs may impact on one or more levels of the biological processes influencing a trait, including DNA methylation, gene expression, protein synthesis, cellular functioning, ‘house‐keeping’ mechanisms within the neuronal microenvironment, system‐level brain morphology and functioning and environment. GWAS output reflects the final endpoints of a ‘mixture’ of many biological processes. Groups of SNPs may share an involvement in the same biological pathways, which can be reflected in their covariation of effect sizes across different brain traits. Exploiting the high dimensional and pleiotropic nature of brain MRI‐GWAS, we introduce a novel, multivariate method that can help to translate the GWAS signal of multiple traits into more interpretable factors. These factors could provide novel insight into the shared biological mechanisms across brain structures, tissues, and/or imaging modalities. We propose genomic independent component analysis (ICA) and genomic principal component analysis (PCA) applied to the GWAS summary statistics of thousands of structural and functional brain imaging derived phenotypes (IDPs), to identify hidden (i.e., latent) genomic factors influencing brain structure and function.

Alternative approaches for investigating pleiotropic genome‐wide associations have been considered to identify hidden patterns in large GWAS. Genomic structural equation modelling (SEM) tests the fit of a priori defined latent factors to up to a few dozen phenotypes^16^ and has been applied successfully to psychiatric^16^, ^17^, ^18^ and cognitive traits.^19^ Another new method, Multivariate Omnibus Statistical Test (MOSTest) integrates multiple phenotypes by combining each SNP's test‐statistics across traits in a manner akin to meta‐analysis.^20^ Other recent work has applied PCA to genome‐wide variant effect‐sizes among multiple phenotypes to derive a number of latent genomic factors.^21^, ^22^ Here, we introduce an approach to simultaneously recover the most prominent principal as well as independent components across thousands of traits. This data‐driven method can identify specific genetic factors that modulate distinct sets of phenotypes within the analysis.

PCA and ICA are powerful decomposition methods to reduce the dimensionality of a large number of observations into fewer, often more interpretable components that capture covariation patterns across observations.^23^ PCA finds orthogonal components in the data that capture variance consecutively, with the first principal component (PC) capturing maximum variance, and the second and subsequent ones capturing variances orthogonal to the previous ones. ICA on the other hand is an unsupervised source separation method that decomposes a complex signal into its constituent maximally independent parts, assuming a linear combination of (non‐Gaussian distributed) signal, structured noise and Gaussian distributed stochastic noise. ICA thus maximises independence between the components while allowing the component weights to be non‐orthogonal if needed, which makes it more suitable to recover distinct sources of signal from noisy data that can be mixed within and across the initial variables (in our case, SNPs).^24^ Thus, compared to PCA, ICA captures variance less efficiently, but is designed to ‘unmix’ a complex signal into its constituents or sources, which become more visible and interpretable as a consequence.^25^ If we interpret current high‐dimensional MRI‐GWAS data as a signal composed of multiple underlying generative mechanisms, the latent genomic sources of variation in GWAS output may reflect distinct biological pathways. Genomic ICA is therefore based on the premise that the genetic variants influencing the same biological processes will impose more similar associations across thousands of brain IDPs, while SNPs associated with distinct biological processes have different patterns of associations across IDPs. While this form of latent structure of genomic effects has been explored previously in expression data,^26^, ^27^ it has not been applied to genome‐wide allelic effects on polygenic traits or had its reproducibility tested. We posit that genomic PCA and ICA may furthermore capture genomically distributed components that reflect consistent effects that are more environmentally mediated but have nevertheless been shown to be heritable, such as sociodemographic metrics or lifestyle factors.^17^, ^28^

In the present paper, we present genomic PCA and ICA as novel, fully data‐driven methods to decompose large, high‐dimensional GWAS summary statistics. This work has evolved from our previous pilot work^29^ and is therefore based on the same underlying rationale. In the present study, we present for the first time our complete and final methodological approach of genomic ICA, extended with PCA, along with a systematic evaluation of the robustness under a multitude of different analytic parameters, dimensionality of the output and other methodological considerations. We test our methods on the GWAS output of 2240 brain MRI traits from the UK Biobank (UKBB), with large, non‐overlapping discovery (*n* = 22,138) and replication (*n* = 11,086) samples.^8^ We determine the reproducibility for multiple versions of our method with varying analytic parameters. We evaluate the output at multiple dimensions (numbers of components). We also apply genomic PCA and ICA to both raw univariate GWAS SNP effect betas and *z*‐transformed GWAS SNP effect betas. Lastly, we provide a head‐to‐head comparison of genomic ICA and genomic PCA reproducibility with the reproducibility of corresponding univariate GWAS that was used as input. This work demonstrates the robustness of genomic PCA and ICA to decompose GWAS signal capturing hidden genomic sources of individual differences in variation across thousands of heritable brain traits.

## 2 METHODS

### 2.1 Data

We acquired GWAS summary statistics from the Oxford Brain Imaging Genetics (BIG‐40) database. BIG‐40 contains the results of over 4000 GWASs that were performed using brain imaging (MRI) derived phenotypes (IDPs) in the UKBB consortium.^30^ For this study, two releases of GWAS summary statistics from the UKBB, with 11,086 (11 k) and 22,138 (22 k) participants, respectively, were retrieved.^8^ These samples are non‐overlapping and contain an identical set of IDPs, making them well suited for discovery and replication. Due to low heritability of node‐to‐node functional connectivity metrics,^1^ we excluded these from the analysis, leaving 2240 IDPs for further analysis. Notably, the amplitudes of functional signal fluctuations and six ICA‐derived ‘global’ measures of functional connectivity were included, as they were shown to be heritable.^1^, ^8^ Other IDPs included metrics from classes of T1‐weighted MRI, diffusion MRI, susceptibility‐weighted imaging (SWI), fluid‐attenuated inversion recovery (FLAIR), task MRI and quality control (QC) procedures. For details on the GWAS pipeline of the UKBB, please refer to the main publications^1^, ^8^ and the website including these UKBB releases (https://open.win.ox.ac.uk/ukbiobank/big40).

### 2.2 Clumping

We applied genome‐wide SNP clumping to reduce local SNP dependencies stemming from linkage disequilibrium (LD). First, we pruned all available SNPs with a threshold of *r* ^2^ < 0.3. Then, we considered the smallest *p*‐value of each SNP across the GWASs of 2240 brain IDPs for clumping. A genomic window size of one mega base, a lead variant *p*‐value threshold of 10^−5^ and a LD‐threshold of *r* ^2^ >0.1 were used as clumping parameters. LD was estimated in a random subsample of 10,077 Caucasian UKBB participants (data field number 22006). This procedure reduced the total number of genome‐wide SNPs from *n* = 17,103,079 to 157,893. Thereby, we minimise the impact of LD on local SNP‐to‐SNP correlations while keeping brain‐related lead variants within each LD block. This clumping procedure is in line with the consensus in the field, whereby we deem within‐chromosome lead‐SNPs associated with genetic loci as independent at *r* ^2^ < 0.1.^31^ To make the methodology more sensitive to genetic effects of common neurological and psychiatric disorders for future downstream analyses, we supplemented the *n* = 157,893 clumped SNPs with additional lead SNPs associated with attention deficit/hyperactivity disorder (ADHD) and Alzheimer's disease (AD), thereby introducing an additional *n* = 7471 SNPs into the analysis. These lead SNPs were derived from clumped GWAS summary statistics on ADHD^32^ and AD.^33^ The summary statistics were clumped with *r* ^2^ < 0.3 and a minimum *p*‐value threshold of *p* < 0.001. This increased the number of SNPs from *n* = 157,893 to 165,364.

### 2.3 Genomic ICA and genomic PCA

We concatenated all GWAS regression values, representing the genome‐wide SNP effect sizes, across all IDPs, generating an *m* × *n* brain‐wide genome‐wide matrix of imaging IDPs (*m* = 2240) and genetic variants (*n* = 165,364 clumped SNPs). Subsequent multivariate decomposition was applied to two versions of the *m* × *n* brain‐wide genome‐wide matrix: the raw GWAS betas and the *z*‐transformed GWAS betas, quantifying SNP effect sizes standardised for their own standard errors. We decomposed the brain‐wide genome‐wide matrices of SNP effect sizes using v3.15 of Multivariate Exploratory Linear Optimised decomposition into independent components (MELODIC), which is a probabilistic ICA algorithm maximising non‐Gaussianity in the reconstructed independent sources.^34^ In the standard pipeline, MELODIC starts by applying probabilistic PCA to the data, thereby decomposing the data into a predefined number of principal components. Then, the algorithm rotates these principal components to optimise a measure of independence (i.e., non‐Gaussianity), thus producing the same number of independent components. Each extracted component consists of a latent genomic factor of SNP‐loadings in the SNP dimension and a vector of IDP‐loadings in the MRI dimension. This feature allows us to determine the association of individual IDPs with the corresponding hidden genomic factor. The *m* × *n* brain‐wide genome‐wide matrices were decomposed by MELODIC along the SNP dimension into a maximum of 200 principal and independent components of SNP effect sizes. For further analyses, dimension 50 was chosen as maximum dimension via visual assessment of the scree plots (Figures 2 and S2) generated from dimension 200, which indicated that dimension 50 provides a good balance between explained variance and model complexity (Figure 2, ~61% SNP effect variance explained in 33 k sample). Here, model complexity refers to the number of components extracted from the data that explain a portion of the variance. Inter‐sample reproducibility was calculated for 5, 10, 25 and 50 dimensions in the discovery (22 k) and replication (11 k) samples for both *z*‐transformed and raw beta input matrices. We disabled the global signal removal option of MELODIC since SNP effect size distribution is already centred on zero under the null assumption, given the random direction of effects dependent on the effect allele. Furthermore, we disabled SNP variance normalisation across IDPs to preserve the magnitude of allelic effects in the genomic components, which contain biological information. This is in contrast to MELODIC applications to fMRI data, where the inherently relative nature of the data warrants the variance normalisation step.^34^ The memory requirements and runtime of the MELODIC algorithm are detailed in the supplement (p. 20). The full software implementation along with the scripts to replicate the present analysis is provided on Github under the following link (https://github.com/LennartOblong/GenomicICA).

### 2.4 Reproducibility testing

To determine reproducibility of principal and independent genomic sources in independent samples across component pairs, we focused on two measures: First, to assess the non‐sparse, global genomic signals of all variants contributing to each component, we calculated Pearson's correlation coefficients of SNP‐wise loadings. The statistical associations were corrected for multiple comparisons by Bonferroni correction for the number for all unique comparisons, here *N* ^2^, where *N* is the number of components per decomposition.

Secondly, to focus only on the sparse part of the genomic sources (the tails of the distribution), SNPs that strongly contribute to each component's multivariate effect relative to the loading distribution, while removing possibly noisy low end of the loadings, we binarized and thresholded the component loadings at values >1. To determine statistical significance of the degree of overlap between each component from the discovery sample with each component of the replication sample, we performed a Fisher's exact test, adjusting for multiple comparisons by the number of contingency tables generated. Fisher's exact test is exact under the assumption that lead‐SNPs with *r* ^2^ < 0.1 are statistically independent, such that the expected degree of overlap under the null can be calculated. To assess if genomic components are robust to the inclusion of low‐reproducibility univariate GWAS IDPs, we performed a post‐hoc correlation analysis between the vector of univariate GWAS reproducibility correlation coefficients and the component loadings in IDP‐space. Given some degree of LD was still present (at *r* ^2^ < 0.1), we also derived the number of effectively independent SNPs^35^ to determine if our analysis should be adjusted to account for this slight remaining dependence between lead‐SNPs. The method and the outcome of this analysis are described in the supplement (p. 1 and 2). From this supplementary analysis, we concluded that the difference between the number of effective (independent) SNPs given our LD threshold and the actual number of SNPs was negligible.

### 2.5 Reproducibility of univariate GWAS

We determined reproducibility of raw, univariate GWAS SNP effect sizes and *z*‐transformed, univariate SNP effect sizes separately to provide a benchmark for the decompositions of both versions of input data. We computed the Pearson's correlation coefficient (*r*_SNP_) of variant effect sizes across independent samples. Maximum *r* ^2^_SNP_ is theoretically determined by additive SNP heritability (*h* ^2^_SNP_) of each trait and provides a benchmark for assessing the reproducibility of PCA and ICA genomic components.

### 2.6 Visualisation of IDP clusters and SNP loadings of genomic components

The decomposition of large MRI‐GWAS data with MELODIC yields a set of IC loadings that quantify the covariation in IDP‐space in correspondence to the covariation of genetic effects in the SNP‐space. The IDP‐space of *N* = 2240 IDPs can be divided into general classes of MRI modalities, namely cortical surface area, cortical thickness, diffusion MRI derived metrics using tract‐based spatial statistics (TBSS) and probabilistic tractography approaches, grey matter volume assessed via FMRIB's Automated Segmentation Tool (FAST), subcortical region of interest (ROI) volume assessed using FMRIB's Integrated Registration and Segmentation Tool (FIRST), ROI volume across multiple atlases assessed using Freesurfer, task‐based functional MRI and resting‐state functional MRI. To visualise the clustering in IDP‐space driven by genetic effects in SNP‐space, we embedded the IDP‐loadings into a two‐dimensional space using t‐distributed stochastic neighbour embedding (t‐SNE).^36^ To maximise the clustering potential of t‐SNE, the visualisation was performed on the decomposition of the combined sample of 11 k and 22 k UKB samples. To visualise the component SNP loadings, we constructed Manhattan‐like plots for the components derived from the same combined sample. To determine the significance level of each SNP‐loading within each component, we calculated the cumulative distribution function for each of the SNP loadings with respect to the mean and standard deviation of the component. Once obtained, we created Manhattan‐like plots for each of the components, visualising the contributions of loci across the genome.

## 3 RESULTS

### 3.1 Raw and *z*‐transformed univariate GWAS

**FIGURE 1:**
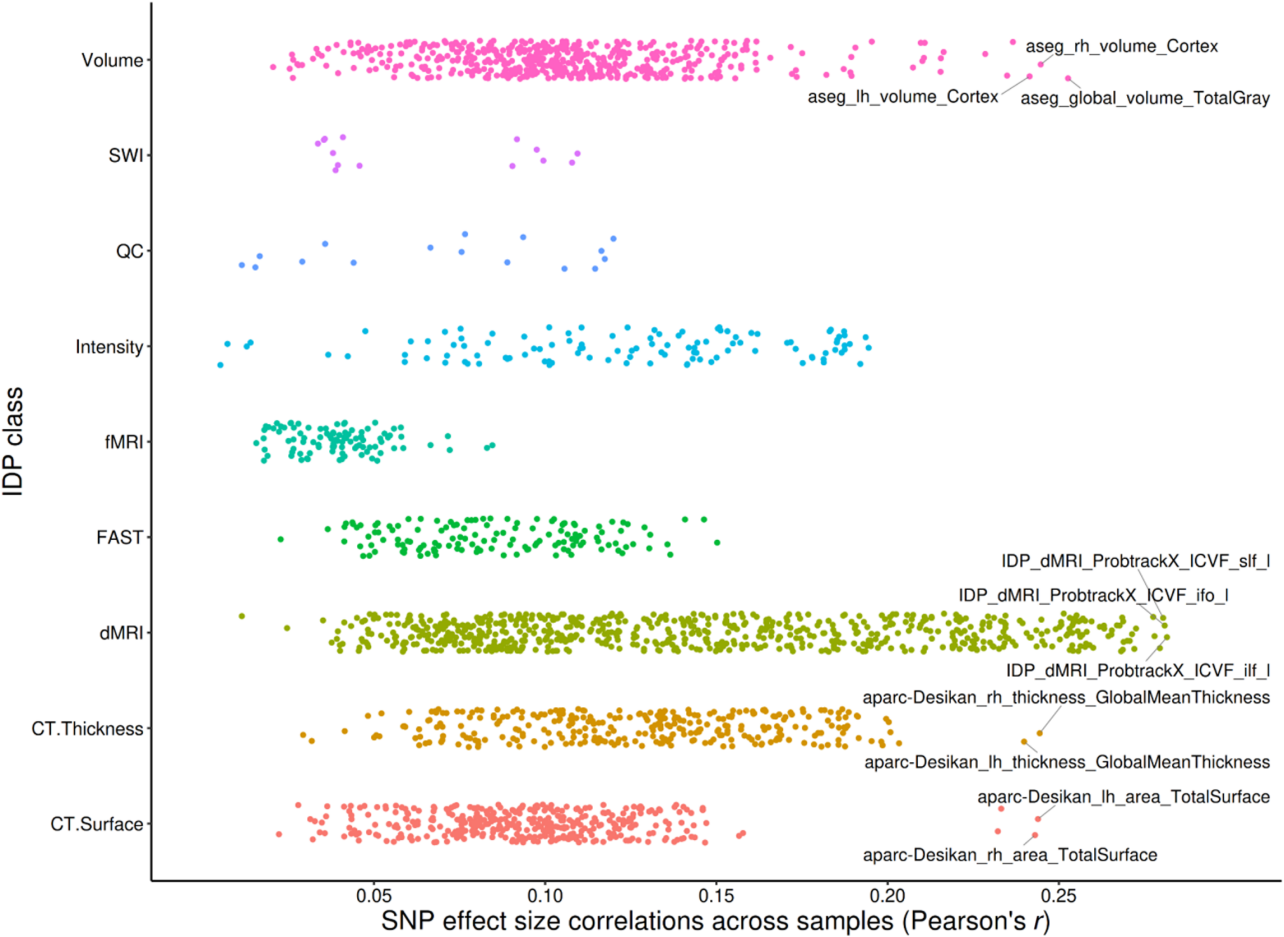
Reproducibility of the *z*‐transformed, univariate genome‐wide association study (GWAS) single nucleotide polymorphism (SNP) effect sizes (*n* = 165,364 clumped variants) across independent samples. Reproducibility was evaluated by computing the Pearson's correlation coefficient (rSNP) of the genetic variant effect sizes between univariate GWAS summary statistics across independent samples (11 k sample vs. 22 k sample). Pall < 0.036. For all Pearson's correlation coefficients *r*_SNP_ >0.02, the significance level quickly shrinks (*p* < 6.11*10^−17^). CT, cortical; dMRI, diffusion magnetic resonance imaging; FAST, FMRIB's Automated Segmentation Tool; fMRI, functional magnetic resonance imaging; IDP, imaging‐derived phenotype; QC, quality control; SWI, susceptibility‐weighted imaging.

Reproducibility of *z*‐transformed, univariate GWAS betas across samples ranged from *r*_max_ = 0.28 to *r*_min_ = 0.005 (*r*_mean_ = 0.11). Highest reproducibility was found in phenotypes derived from large white matter tracts derived from *dMRI*, followed by IDPs in global *cortical volume*, *thickness* and *surface area*. *Cortical surface area*, *intensity measures* and *FAST‐derived* measures showed lower reproducibility than *cortical thickness* and *volume*. Lowest reproducibility was found for *SWI* and *fMRI* derived IDPs. Reproducibility of all IDPs is shown in Figure 1. Reproducibility of raw, univariate GWAS beta‐values in terms of Pearson's correlation coefficient, ranged from *r*_max_ = 0.25 to *r*_min_ = 0.003 (*r*_mean_ = 0.09) and was lower than *z*‐transformed univariate GWAS reproducibility. Highest reproducibility was found for similar IDPs as for the *z*‐transformed decomposition and depicted in Figure S1.

### 3.2 Principal genomic components

**FIGURE 2:**
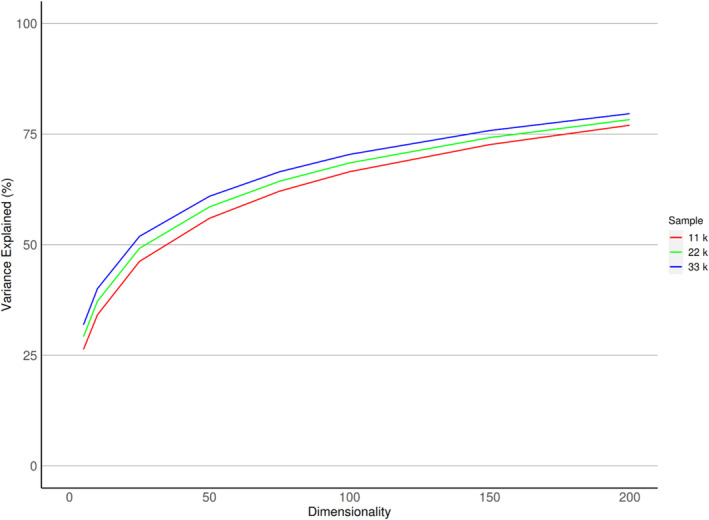
Variance explained by genomic components derived from *z*‐transformed, univariate genome‐wide association study single nucleotide polymorphism effect sizes at principal component analysis dimensions 5, 10, 25, 50, 100, 150 and 200.

**FIGURE 3:**
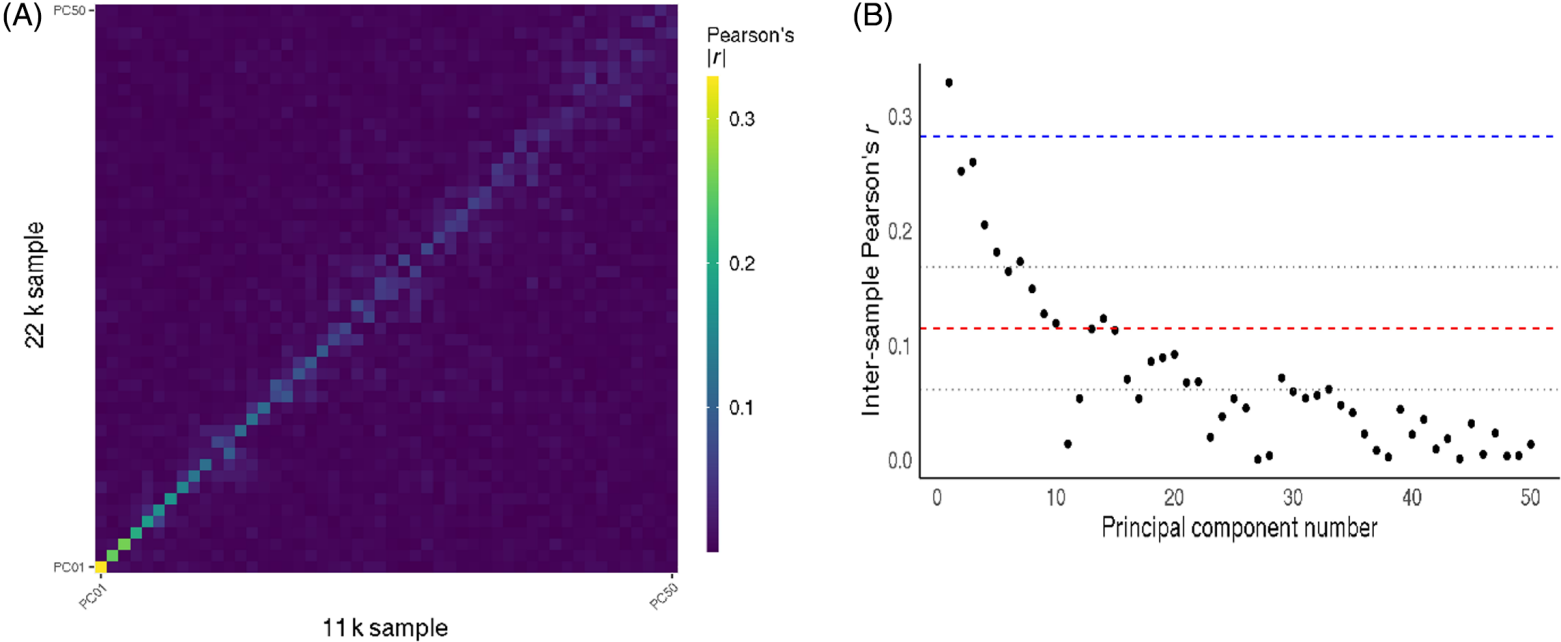
(A) Inter‐sample reproducibility of principal genomic components (PC) derived at dimension 50, from *z*‐transformed univariate genome‐wide association study (GWAS) single nucleotide polymorphism effects, displayed as the Pearson correlation coefficient. (B) The maximum reproducibility per principal component as a scatterplot, with the Pearson correlation coefficient on the *y*‐axis. The red dashed line denotes the mean of raw, univariate GWAS reproducibility, with the grey, dotted lines indicating one standard deviation around the mean. The blue dashed line indicates the maximum reproducibility of *z*‐transformed, univariate GWAS betas.

The first five PCs derived from *z*‐transformed GWAS captured 31.9% of the variance across SNP effect sizes, while decomposing into 200 PCs increased the variance explained to 79.6% (Figure 2). A nearly identical pattern was found for the variance captured by the raw GWAS betas (Figure S2). Inter‐sample reproducibility of PCs at dimension 5 was high, ranging from |*r*_max_| = 0.33 (*p*_adj_ = <10^−308^) to |*r*_min_| = 0.18 (*p*_adj_ = <10^−308^), with decreasing reproducibility at higher dimensions (Figure 3A,B; Table S1). Notably, the first PC was more reproducible than the maximum reproducibility across all 2240 univariate *z*‐transformed GWAS outputs (Figure 3B). The first ten PCs derived from *z*‐transformed GWAS all showed higher reproducibility than mean reproducibility of univariate *z*‐transformed GWAS (Figure 3A,B). Subsequent PCs #11‐#50 successively explained less variance and were also less reproducible across independent samples (Figure 3B). The reproducibility of PCs derived from raw GWAS data overall showed a similar pattern but was generally lower than the PCs derived from *z*‐transformed GWAS data (Table S1; Figure S3). The correlation analysis to test if PC IDP‐loadings are robust to IDPs of low reproducibility showed that PC1 correlates strongest with univariate IDP‐GWASs (*r* = 0.7, *p* = <10^−308^), followed by PC2 (*r* = 0.43, *p* = 4.14*10^−101^). Correlations for the subsequent components were lower (Table S4).

### 3.3 Independent genomic components

**FIGURE 4:**
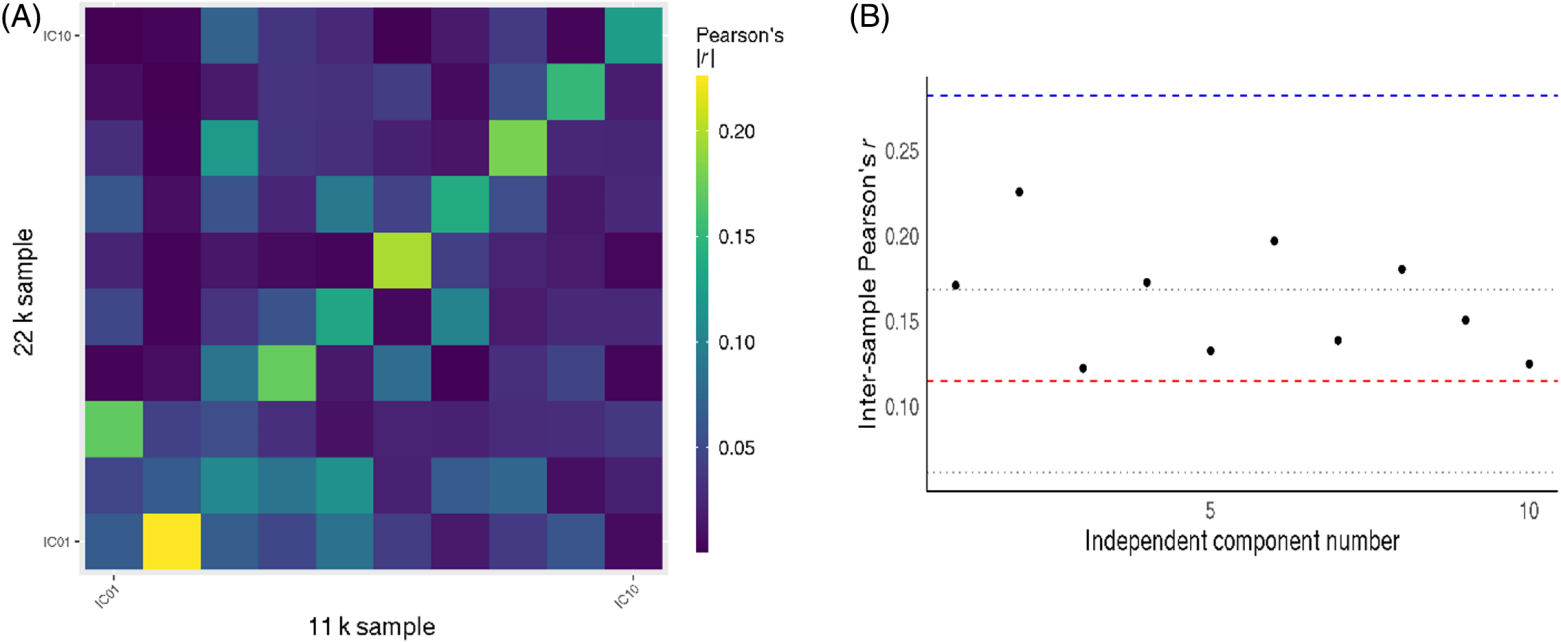
Inter‐sample reproducibility of independent genomic components (IC) derived at dimension 10 from *z*‐transformed univariate genome‐wide association study (GWAS) single nucleotide polymorphism effects (A). (B) The maximum reproducibility per independent component derived from *z*‐transformed univariate GWAS as a scatterplot, with the Pearson correlation coefficient on the y‐axis. The red dashed line denotes the mean reproducibility of the respective univariate GWAS, with the grey, dotted lines indicating one standard deviation around the mean. The blue dashed line indicates the maximum reproducibility of the respective univariate GWAS betas.

ICs derived from *z*‐transformed, univariate GWAS likewise showed highest reproducibility at dimension 5 (|*r*_max_| = 0.25, *p*_adj_ = <10^−308^; |*r*_min_| = 0.15, *p*_adj_ = <10^−308^; |*r*_mean_| = 0.20; Figure S3A,B). Reproducibility dropped with increasing dimensionality (Figures S4 and S5). Improved reproducibility compared to univariate GWAS was found up to dimension 10 (|*r*_max_| = 0.23; |*r*_min_| = 0.12; |*r*_mean_| = 0.16; Figure 4A,B). At dimension 10, we found that all components from the discovery sample correlated with either one or multiple replicated components with high statistical significance (0.23 > |*r*| > 0.10; *p*_all_ = <10^−308^; Table S2). ICs derived from *z*‐transformed GWAS data were more reproducible than ICs derived from raw GWAS data (Table S2). While the maximum reproducibility among the 10 ICs was lower than the maximum univariate reproducibility among 2240 IDPs (|*r*_IC2_| = 0.23 vs. *r* = 0.28), all 10 ICs exceeded mean univariate reproducibility (Figure 4B). After binarizing, the top SNPs of six of the ten independent components of the discovery sample replicated significantly (Fisher's *p*_all_ = <0.003; Table S2). The most strongly correlated IC1 from the discovery sample replicated as IC2 in the replication sample (Fisher's *p*_adj_ = 5.5*10^−66^). Reproducibility statistics of genomic independent components at dimensions 5, 25 and 50 and a comparison with respective, univariate GWAS reproducibility are shown in the supplement (Figures S5–S7). Independent components derived from raw, univariate GWAS followed a similar pattern as the *z*‐transformed decomposition (Figures S4 and S5–S7). The correlation analysis to test if ICs IDP loadings are robust to IDPs of low reproducibility showed that 9/10 ICs follow the expected correlation pattern. IC1 (*r* = 0.64, *p* = 1.1*10^−255^) and IC4 (*r* = 0.63, *p* = 5.45*10^−250^) show strong correlations with the univariate reproducibility vector, suggesting that they are driven by highly reproducible univariate IDP effects. Other components display moderate to weak correlations (0.40 > *r* > 0.11), even though most are highly significant (8.4*10^−87^ < *p* < 7.7*10^−8^) (Table S3).

### 3.4 IDP clustering and SNP plots of genomic components

**FIGURE 5:**
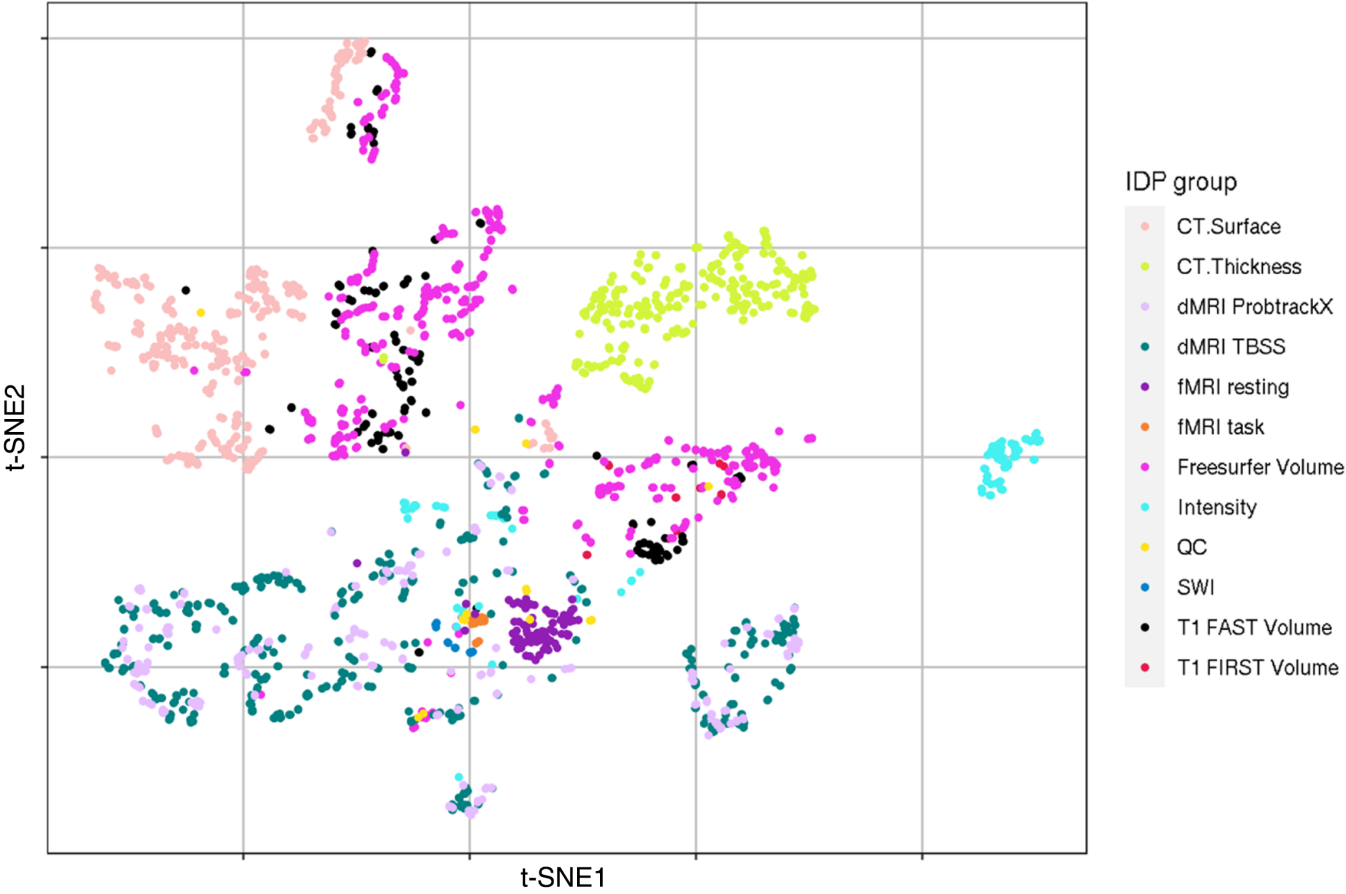
t‐Distributed stochastic neighbour embedding (t‐SNE) based visualisation of imaging‐derived phenotype (IDP) loadings, derived from the decomposition into 10 genomic independent components of *z*‐transformed univariate genome‐wide association study of the combined 11 k and 22 k samples. This plot shows the clustering of IDP loadings across all dimension 10 genomic components, thereby showing the emergence of distinct IDP groups associated with the covariation of specific sets of genetic effects. CT, cortical; dMRI, diffusion magnetic resonance imaging; FAST, FMRIB's Automated Segmentation Tool; FIRST, FMRIB's Integrated Registration and Segmentation Tool; fMRI, functional magnetic resonance imaging; QC, quality control; SWI, susceptibility‐weighted imaging.

Based on the reproducibility analysis (Figures 3 and 4), in combination with the scree plot (Figure 2), we concluded that the decomposition of *z*‐transformed, univariate GWAS at dimension 10 was optimal in terms of variance explained, model complexity (i.e., number of components generated) and inter‐sample reproducibility among the decomposition parameters tested. The t‐SNE analysis of the corresponding IDP loadings clearly showed clustering of IDP loadings along the boundaries of larger IDP groups in MRI modalities (Figure 5). These results indicate distinct associations of concerted genetic effects on traits from different modalities and similar genetic effects within imaging modalities (e.g., cortical thickness and cortical surface area vs. dMRI measures). In some cases, different methods used to derive metrics related to similar modalities resulted in the splitting into different clusters, such as with T1‐weighted images of cortical thickness and surface area, SWI and MRI intensity IDPs. Different methods in diffusion MRI did not follow this trend, and probabilistic tractography derived IDPs and TBSS‐derived IDPs showed clustering according to similar genetic associations.

The visualisation of SNP loadings showed that the components are driven by diverse locus ‘structures’ that include strong single locus (IC10, Figure S15), double locus (IC7, Figure S12) and multiple loci (IC1, Figure S6). Most of the PCs and ICs are driven by multiple loci across the genome, and all Manhattan‐like plots are shown in the supplemental material (Figures S7–S27).

## 4 DISCUSSION

GWAS analyses of the past decades have enhanced our understanding of common genetic variants influencing imaging derived brain phenotypes, but pleiotropic and polygenic effects, paired with small effect sizes of genetic variants, limit the mechanistic interpretability of GWAS data. Genomic PCA and ICA leverage pleiotropy and polygenicity through the assumption that GWAS effect sizes across genetically correlated traits are a linearly mixed signal containing structured and Gaussian noise. This makes these methods well suited to uncover hidden genomic structure within large GWAS summary statistics. This could enhance our understanding of how genes act.

Here, we decomposed high‐dimensional neuroimaging GWAS data, without a priori assumptions, into a smaller set of multivariate principal and independent components. Genomic components show moderate reproducibility across independent samples, substantially improving upon mean reproducibility of univariate GWAS. For both raw and *z*‐transformed betas, PCA captured most of the variance within the first three components (~26.5%), with the following components capturing less variance. Reproducibility of the first independent genomic components was lower than that of the principal components, but at dimension 10, all ICs also showed improved reproducibility across all IDPs compared to mean reproducibility of respective GWAS data. This enhanced stability of the components genetic loadings relative to the raw GWAS data holds promise for future applications of genomic PCA/ICA in genomic data analysis, and for improved downstream analyses such as identifying gene‐sets, more accurate polygenic score prediction, or other downstream analyses. Further, our results demonstrate that IDPs from distinct MRI modalities show clear clustering patterns (Figure 5) captured by the genomic components. These patterns indicate distinct MRI‐modality‐specific and tissue‐specific genetic effects.

The SNP loadings of genomic components showed that the components can be driven by strong single‐, double‐ or multiple loci. Most components are driven by multiple loci across the genome, which indicates that distant parts of the genome have a concerted effect on brain traits.

The majority of the IC IDP loadings correlate strongly and significantly with the reproducibility estimates of the univariate IDP‐GWASs. This indicates that the ICs were predominantly driven by high‐reproducibility IDP GWAS signals. For the PCs, the first PC correlates most strongly with the univariate reproducibility, with the correlations quickly dropping in subsequent components. These findings are consistent with the theoretical assumptions that the first PC will capture the largest amount of variance across all IDP‐GWAS summary statistics, thereby the most reproducible IDPs are largely captured by PC1.

Reproducibility of genomic PCs and ICs derived from *z*‐transformed data was higher than those of components derived from raw beta‐values. This is likely because the *z*‐transform accounts for the variable standard error around the SNP‐betas making them less sensitive to noisier SNP estimates with large standard errors, which would be especially the case for low‐MAF SNPs. With increasing discovery sample sizes for GWAS, the standard errors shrink, ultimately leading to convergence of raw and *z*‐transformed decompositions to identical results. Increases in discovery sample sizes will also enable decompositions into more and more reliable independent sources of genomic signal, thereby increasing the sensitivity of genomic PCA and ICA to uncover more refined and likely more specific clusters of genetic effects on sets of brain features.

The present work demonstrates that genomic PCA and ICA components capture IDP‐group specific, genomic signal that is stable across independent samples and robust to low‐reproducibility IDPs. Further, the data is decomposed without a priori assumptions and can include thousands of phenotypic traits. Theoretically, this method is not limited to specific data types. It can be used on fMRI data^34^ and, as demonstrated here, on genomics data to extract hidden independent sources of relevant signal. Furthermore, the more data is available for ICA to parse, the better the potential signal in the data can be separated from structured noise. As such, ICA thrives on more potential signals hidden in data, as long as the structured noise permeates the same data. This makes a case for including non‐MRI based GWAS data in genomic PCA and ICA decompositions as it would further improve discoverability of more fine‐grained sources of genetic variation from large GWAS. Univariate GWAS showed highest reproducibility in global cortical morphological and white matter tracts. This aligns with prior studies favouring large white matter structures.^37^ This implied that GWAS benefits from ‘signal averaging’ across smaller IDPs, which reduces noise and enhances reproducibility. Genomic PCA and ICA similarly boost signal‐to‐noise ratios by aggregating shared signals within the same component, as it does in neuroimaging applications.^38^ The flexibility of genomic PCA and ICA could also be applied to epigenome‐wide and transcriptome‐wide data, thereby deepening our understanding of environmental effects on genetic expression patterns. Additionally, future research could explore other, powerful means of decomposition that are sensitive to weightings of included modalities by decomposing across data domains using, for example, linked ICA^39^ or SuperBigFlica.^40^ Another avenue that we currently pursue is the computation of individualised component scores, following the polygenic risk score framework, to stratify existing cohorts for normative modelling and personalised medicine. Investigations into the alignment of component IDPs with gene‐sets based on cell specific gene expression, molecular pathways, or brain homeostasis will reveal the potential of our methods.

### 4.1 Limitations

Association of GWAS SNP effects decomposed with genomic ICA may still influence one another on any level of the causal chain from DNA molecule to fully developed brain IDP, and thereby may conceal effects. This touches on the question of what noise means in the context of genetic data. While genomic PCA and ICA are well suited to extract structured noise from data and capture it in individual components, we cannot be certain how this structured ‘noise’ affects brain development on any level of biological, causally related mechanisms. Some components might capture structured noise insufficiently captured by quality control protocols, such as population stratification, assortative mating, or cryptic relatedness. Other components may capture a mixture of genetic effects related to ‘house‐keeping’ mechanisms that affect brain‐wide mechanisms which influence diverse tissue types and properties. This warrants further analyses of reproducible components using, for example, gene‐set enrichment and other bioinformatics tools.

The current implementation of genomic ICA relies on clumping of GWAS summary statistics to reduce the number of (largely redundant, correlated) SNPs in the analysis, keeping only the strongest SNP associations within each LD‐block. The components can thus achieve a lower genome‐wide coverage than regular GWAS output, with only low LD values. As a result, applicability of the components to certain follow‐up analyses, such as LD score regression, is limited. This warrants testing of summary statistic imputation methods to increase genome covarge of the components, and future applications of genomic ICA to GWAS summary statistics with a less stringent clumping paradigm to potentially broaden its suitability for other follow‐up analyses.

Even though we decompose a large matrix containing ~2000 brain IDPs, the inclusion of non‐MRI based data may further improve the outcome of genomic ICA by providing the method with more data to parse, as discussed above. This is different from the approach used by Fürtjes et al., which is closest to our proposed methods here, where only structural MRI derived volumetric IDPs were used.^21^ While the application of PCA is common between their and our work, this constraint in the IDP space might have limited their discoverability of distinct genetic components that are shown to map to distinct modality clusters (Figure 5). This shows the potential of using data‐driven, hypothesis‐free methods on large GWAS‐MRI data to uncover hidden structure across IDPs. The inclusion of non‐MRI based data in addition to the ~2000 brain IDPs may reconstruct yet more sources of genomic variation related to behavioural measures, disease biomarkers and psychiatric conditions. Further, the present analysis using GWAS data is the Western European‐centric ancestry of the UKBB sample. These components may be more or less sensitive to genetic effects specific to genetic ancestries and socio‐cultural influences. We recognise the efforts to expand GWAS to international cohorts, which will provide more data for these genetic analyses, and we eagerly await the availability of these data to be included into the genomic PCA and ICA framework.

## 5 CONCLUSION

We introduce genomic PCA and ICA as a novel method to decompose large MRI‐GWAS summary statistics to efficiently and reproducibly extract latent genomic components that affect brain structure and function as measured by MRI. We derived principal and independent genomic sources from a large set of GWAS statistics, containing genetic associations with 2240 brain IDPs. To thoroughly test the efficacy of the method, we decomposed both raw and *z*‐transformed, univariate GWAS SNP effects at multiple component dimensions. Genomic PCA and ICA showed improved inter‐sample reproducibility for both decomposition inputs compared to respective, univariate GWAS SNP effects. Genetic effects captured across components showed clear clustering according to specific MRI modalities and brain features. This makes genomic ICA a promising method to consistently and effectively decompose noisy, brain‐related genome‐wide association data into more reproducible and more interpretable genomic components, capturing covariation of genetic effects across IDPs.

## CONFLICT OF INTEREST STATEMENT

The authors declare no conflict of interest.

## Supporting information


**Data S1.** Supporting information.

## Data Availability

The results of the present study were derived from the following resources available in the public domain: https://open.win.ox.ac.uk/ukbiobank/big40/; https://ctg.cncr.nl/software/summary_statistics; https://pgc.unc.edu/for-researchers/download-results/. The genomic components generated by this study will be made available on Github under https://github.com/LennartOblong/GenomicICA.
