# Supplementary material for: Principal and independent genomic components of brain structure and function
Source: Genes Brain Behav. 2024 Jan 15;23(1):e12876. doi: 10.1111/gbb.12876 (PMC10797248; doi:10.1111/gbb.12876)
Supplement: Supplementary file 1 — Data S1. Supporting information. [file GBB-23-e12876-s001.docx]

Supplemental Material

**Principal and Independent Genomic Components of Brain Structure and Function**

**Additional Analyses**

**Estimation of effective tests**

In the present study we reduced the number of SNPs (n=17,103,079) via clumping and pruning to n=157,893, then enriched the sample with another n= 7,471 SNPs related to Alzheimer’s Disease and ADHD. This was based on pruning with subsequent clumping, yielding lead variants with LD of r^2^<0.1. However, the clumped and enriched variants could still be in LD with each other. To account for this we chose to test the number of effective tests conservatively, to estimate if another round of clumping would be necessary to account for non-independence between SNPs.

The number of effective tests (*M*eff) was estimated based on chromosome-specific correlation matrices to account for full SNP independence, in terms of full linkage equilibrium, between chromosomes, which arises definitionally from the concept of LD. The number of effective tests was estimated following the mathematical framework of Galwey (Galwey, 2009), where we calculated the variance of eigenvalues of SNP-correlation matrices per chromosome, and derived the number of effective tests. This method was chosen above another proposed method that requires the manual setting of the eigenvalue (Gao et al., 2008). Given that we aimed to estimate *M*eff in a data-driven way, we decided to use the Galwey method.

Specifically we *M*eff is defined as
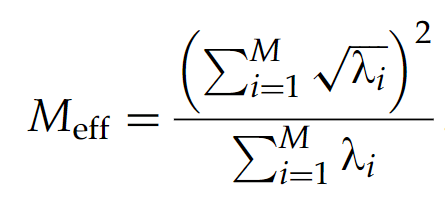


where *M* is the number of tests (or elements in the matrix provided), and lambda denotes the i-th eigenvalues of the matrix given (Galwey, 2009).

To be conservative in light of the inclusion of ~7.000 SNPs post clumping, we assumed correlation matrices, where the diagonal is 1, accounting for full LD of the same SNPs, and with r^2^=0.1 between all remaining SNP pairings. Another consideration was to use an autoregression algorithm to simulate LD between included SNPs. This idea was rejected however, as extracting the actual LD from the 10,077 UKBB individuals would have been less work and computationally more efficient. Then, we calculated the number of effective tests per chromosome, summed the numbers of effective tests per chromosome to arrive at the full number of effective tests for all n=165,364 SNPs. We found that the total number of effectively independent tests, calculated as specified above, was *M*eff=149,919. This reduced the number of tests by n=15,427. Based on this, and the small p-values we observed when calculating Fisher's exact test on the number of SNPs, we determined that the effect was negligible and another round of clumping was unwarranted at this stage.

**Reproducibility of principal and independent genomic components derived from raw GWASs**

Inter-sample reproducibility of PCs derived from raw GWAS betas showed a similar pattern with generally lower reproducibility values compared to the z-transformed components shown in the main manuscript. Please refer to table S1 and figures S3 for details.

Inter-sample reproducibility of ICs was similar, albeit weaker, compared to the components derived from z-transformed GWAS. At dimension 10 eight components exceeded mean reproducibility of univariate GWAS (|r_max_|=0.19, |r_min_|=0.07, |r_mean_|=0.12, figure S4). Components from the discovery sample correlated with one or multiple components in the replication sample with high statistical significance (0.19 > |r| > 0.07; p_all_=<1.88^-157^, table S2). The distribution of component loadings from raw GWAS decompositions were highly kurtotic, which made the investigation of overlap of the component tails unsuitable. Reproducibility of dimensions 5, 25 and 50 and a comparison with raw, univariate GWAS SNP effect reproducibility are shown in the supplement (figure S5 – S7).

# Figures


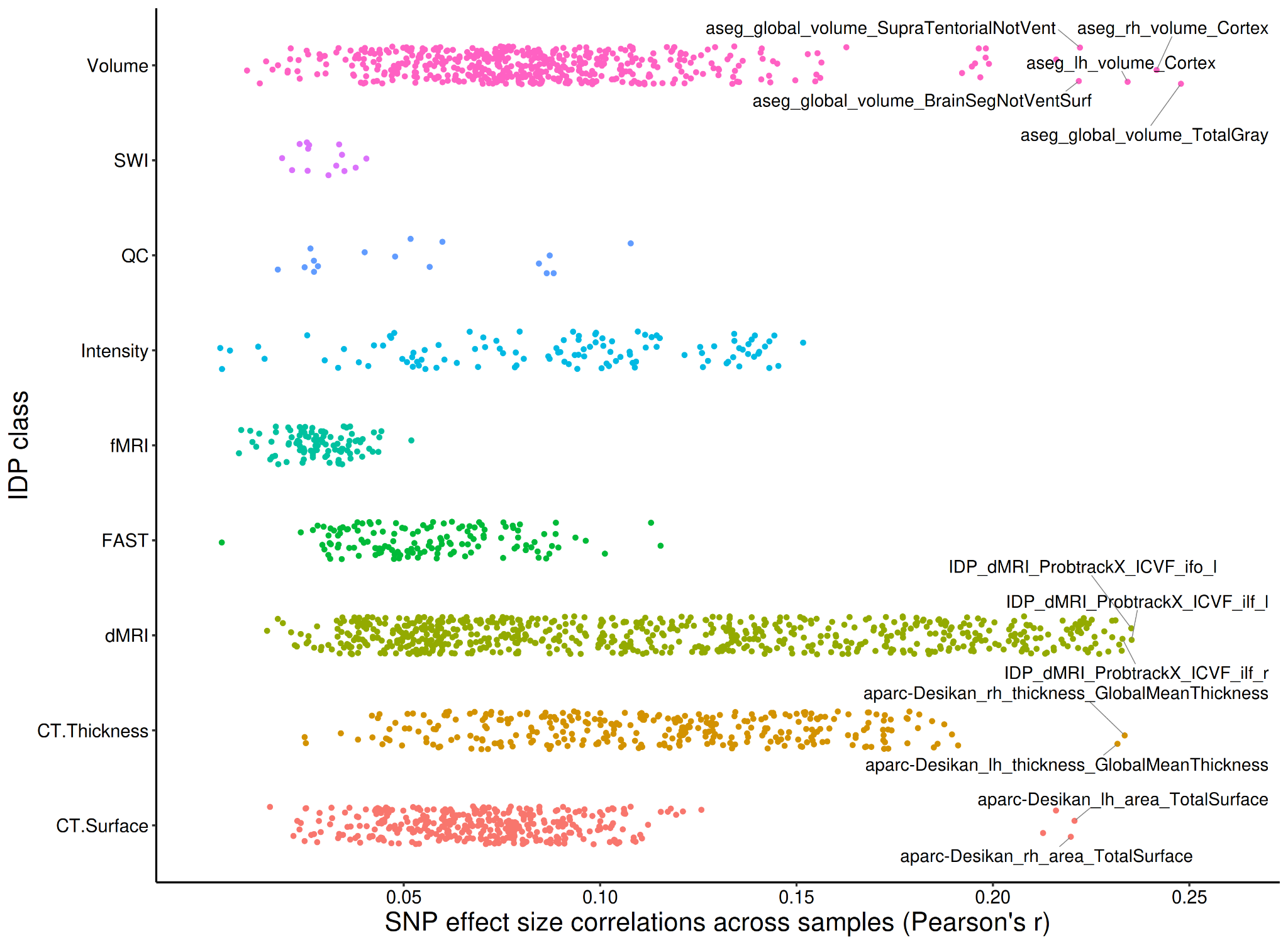


**Figure S1**: Reproducibility of the “raw”, univariate GWAS SNP effect sizes (n=165364 clumped variants) across independent samples. Reproducibility was assessed by computing the Pearson's correlation coefficient (rSNP) of the genetic variant effect sizes between univariate GWAS summary statistics across independent samples (11k sample vs. 22k sample). P_all_ < 0.18. For all Pearson’s correlation coefficients r_SNP_>0.02 the significance level quickly shrinks (p<1.28*10^-16^).


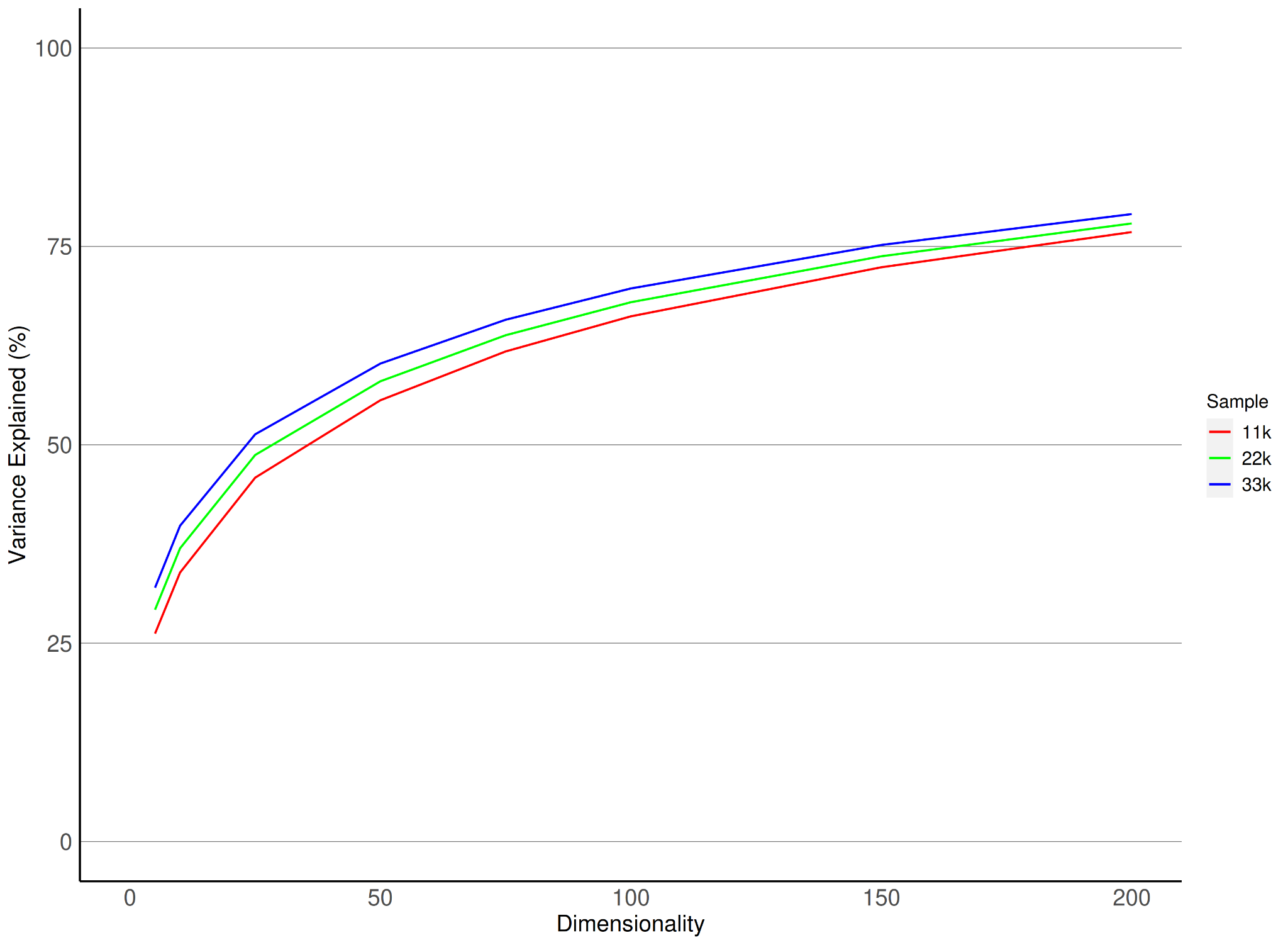


**Figure S2**: Variance explained by genomic components derived from raw, univariate GWAS SNP effect sizes at ICA dimensions 5, 10, 25, 50, 100, 150, and 200.


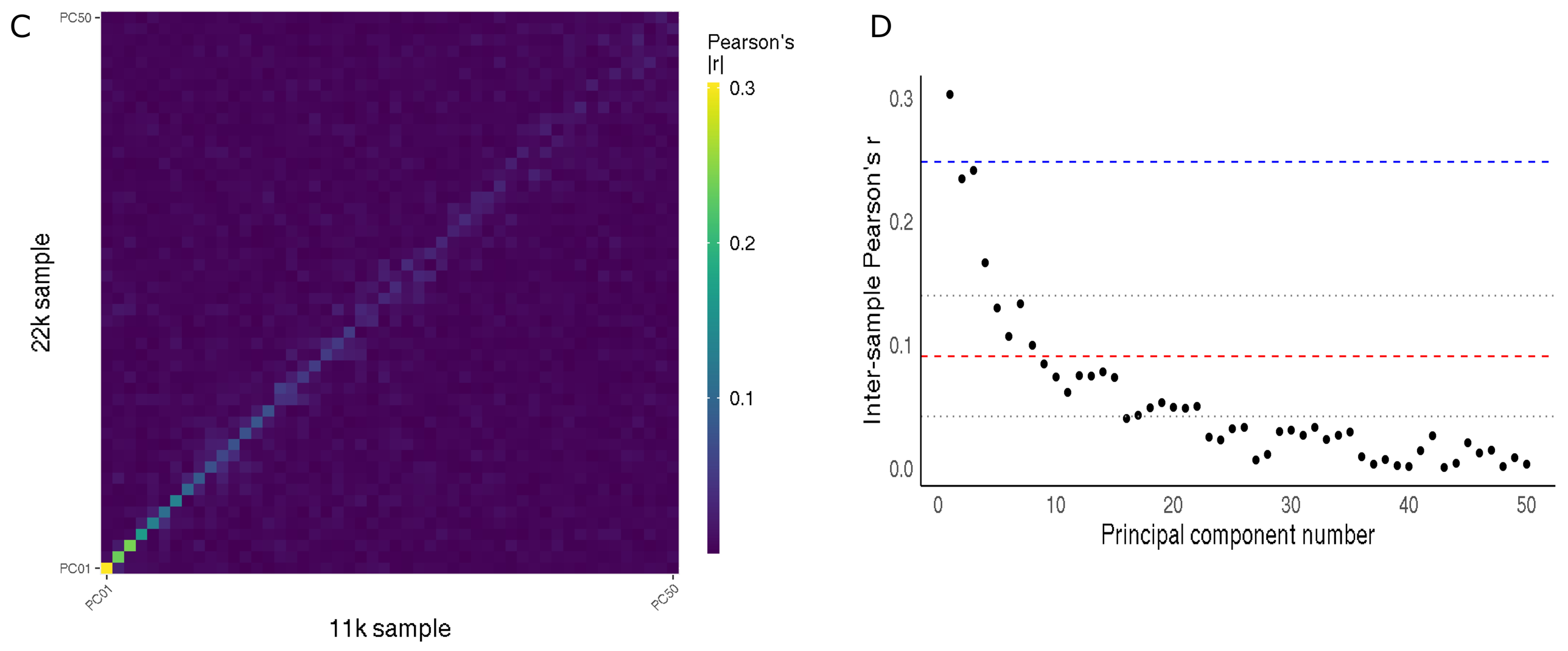


**Figure S3**: Inter-sample reproducibility of principal genomic components derived at dimension 50, from raw-transformed univariate GWAS SNP effects, displayed as the Pearson correlation coefficient. (D) shows the maximum reproducibility per principal component as a scatterplot, with the Pearson correlation coefficient on the y-axis. The red dashed line denotes the mean of raw, univariate GWAS reproducibility, with the grey, dotted lines indicating one standard deviation around the mean. The blue dashed line indicates the maximum reproducibility of z-transformed, univariate GWAS betas.


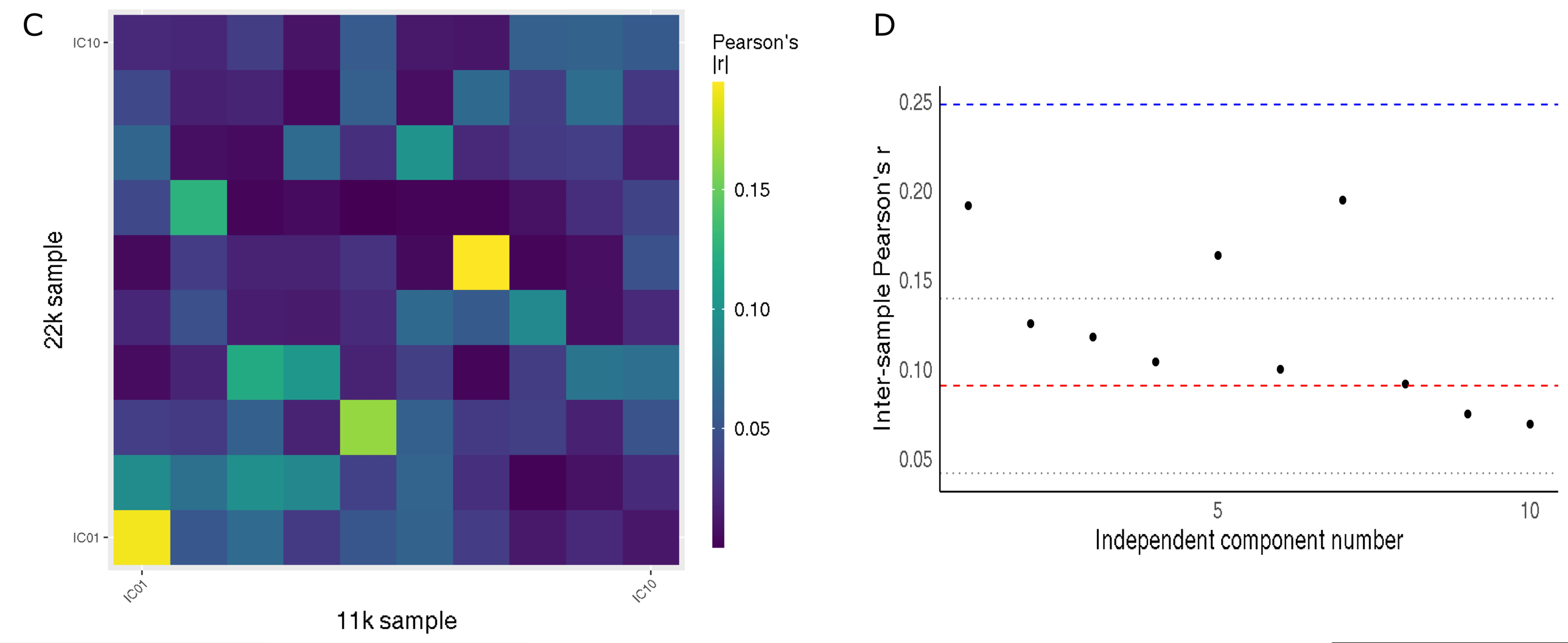


**Figure S4**: Inter-sample reproducibility of independent genomic components derived at dimension 10, from raw-transformed univariate GWAS SNP effects (C). (D) shows the maximum reproducibility per independent component derived from raw-transformed univariate GWAS as a scatterplot, with the Pearson correlation coefficient on the y-axis. The red dashed line denotes the mean reproducibility of the respective univariate GWAS, with the grey, dotted lines indicating one standard deviation around the mean. The blue dashed line indicates the maximum reproducibility of the respective univariate GWAS betas.


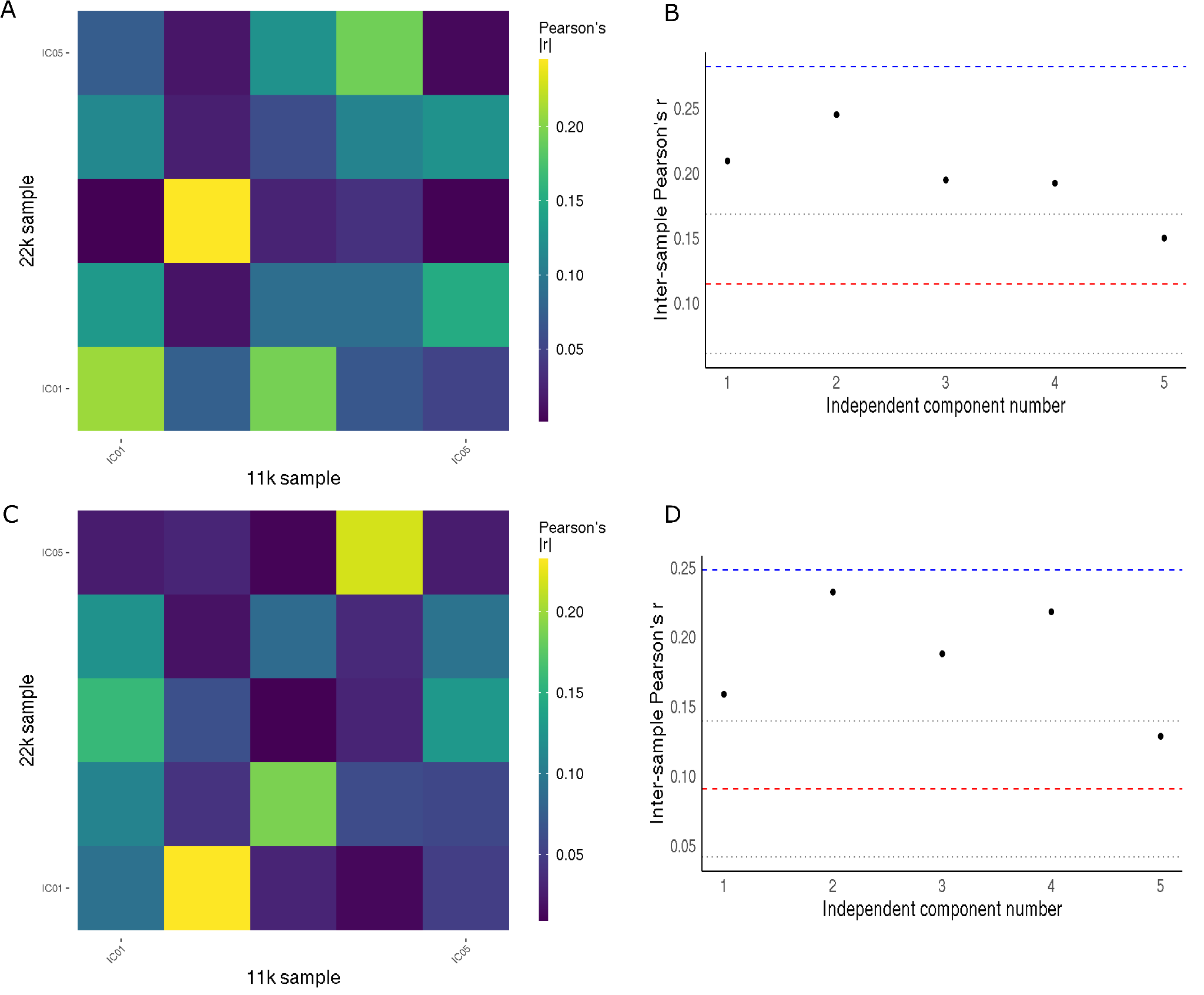


**Figure S5**: Inter-sample reproducibility of independent genomic components derived at dimension 5, from z-transformed (top row) and raw (bottom row), univariate GWAS SNP effects, displayed as the Pearson correlation coefficient in A and C. B and D show the respective reproducibility per independent component as a scatterplot, with the Pearson correlation coefficient on the y-axis. The red dashed line denotes the mean of raw, univariate GWAS reproducibility, with the gray, dotted lines indicating one standard deviation around the mean. The blue dashed line indicates the maximum reproducibility of z-transformed, univariate GWAS betas.


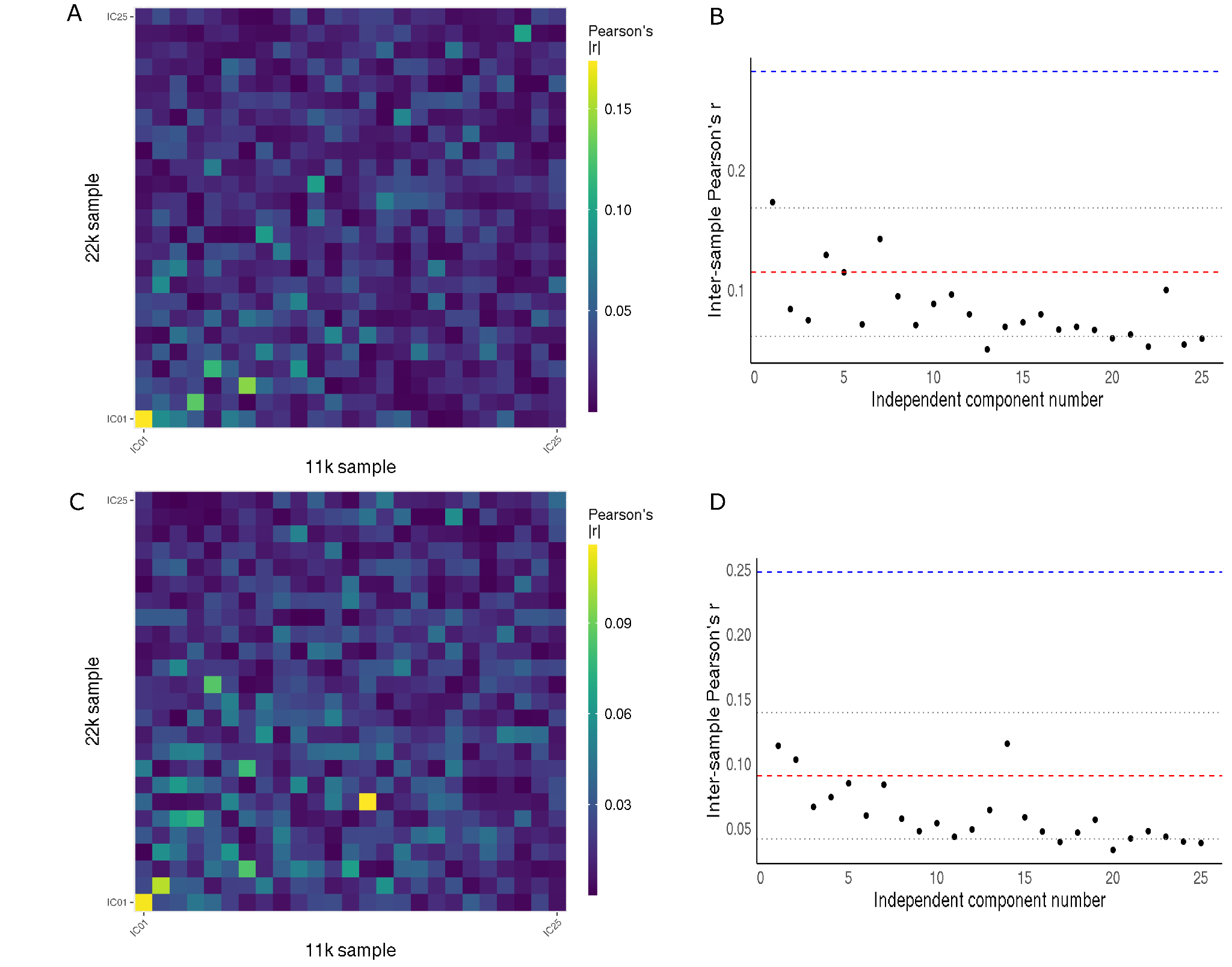


**Figure S6**: Inter-sample reproducibility of independent genomic components derived at dimension 25, from z-transformed (top row) and raw (bottom row), univariate GWAS SNP effects, displayed as the Pearson correlation coefficient in A and C. B and D show the respective reproducibility per independent component as a scatterplot, with the Pearson correlation coefficient on the y-axis. The red dashed line denotes the mean of raw, univariate GWAS reproducibility, with the gray, dotted lines indicating one standard deviation around the mean. The blue dashed line indicates the maximum reproducibility of z-transformed, univariate GWAS betas.


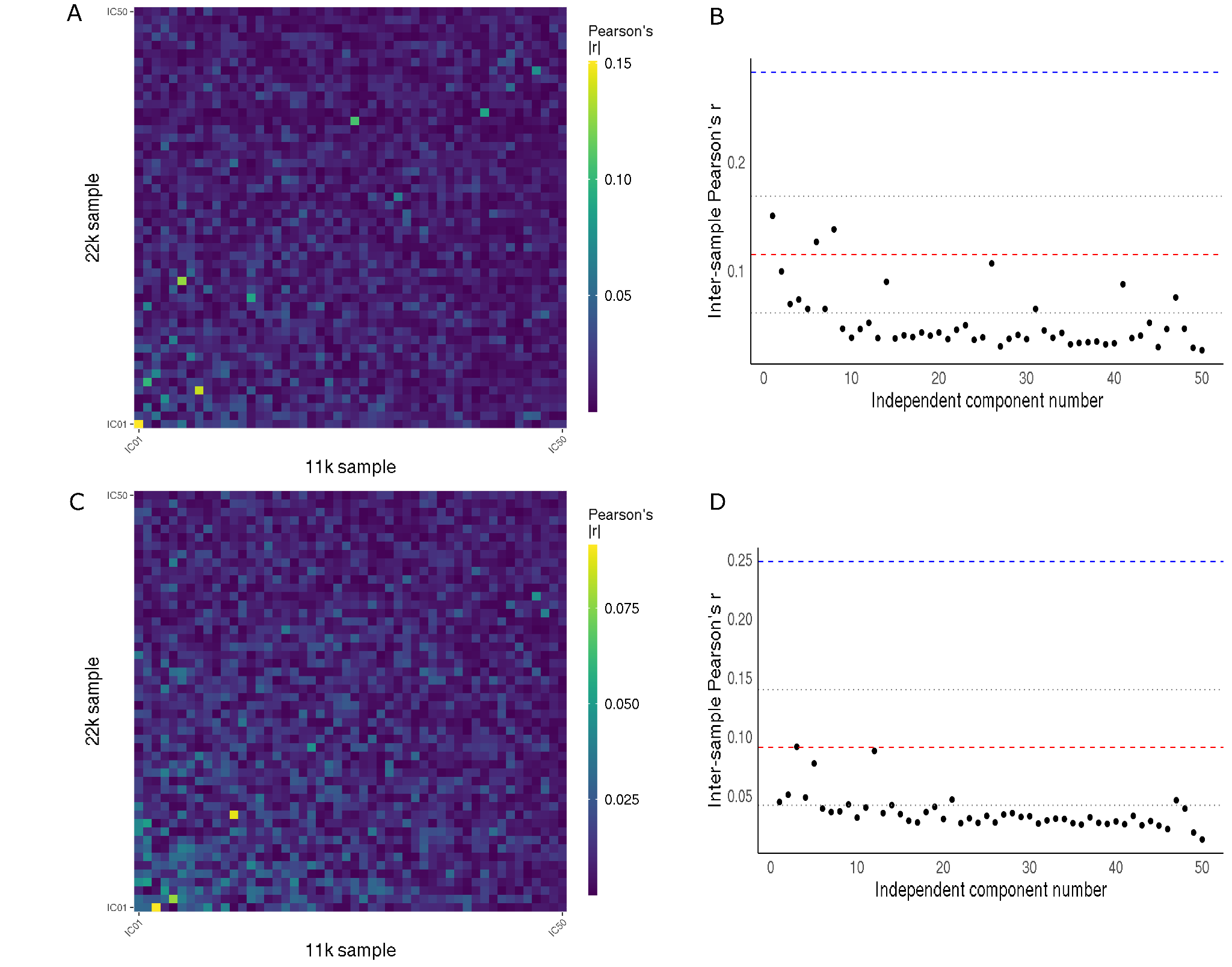


**Figure S7**: Inter-sample reproducibility of independent genomic components derived at dimension 50, from z-transformed (top row) and raw (bottom row), univariate GWAS SNP effects, displayed as the Pearson correlation coefficient in A and C. B and D show the respective reproducibility per independent component as a scatterplot, with the Pearson correlation coefficient on the y-axis. The red dashed line denotes the mean of raw, univariate GWAS reproducibility, with the gray, dotted lines indicating one standard deviation around the mean. The blue dashed line indicates the maximum reproducibility of z-transformed, univariate GWAS betas.

**ICA Manhattan plots**


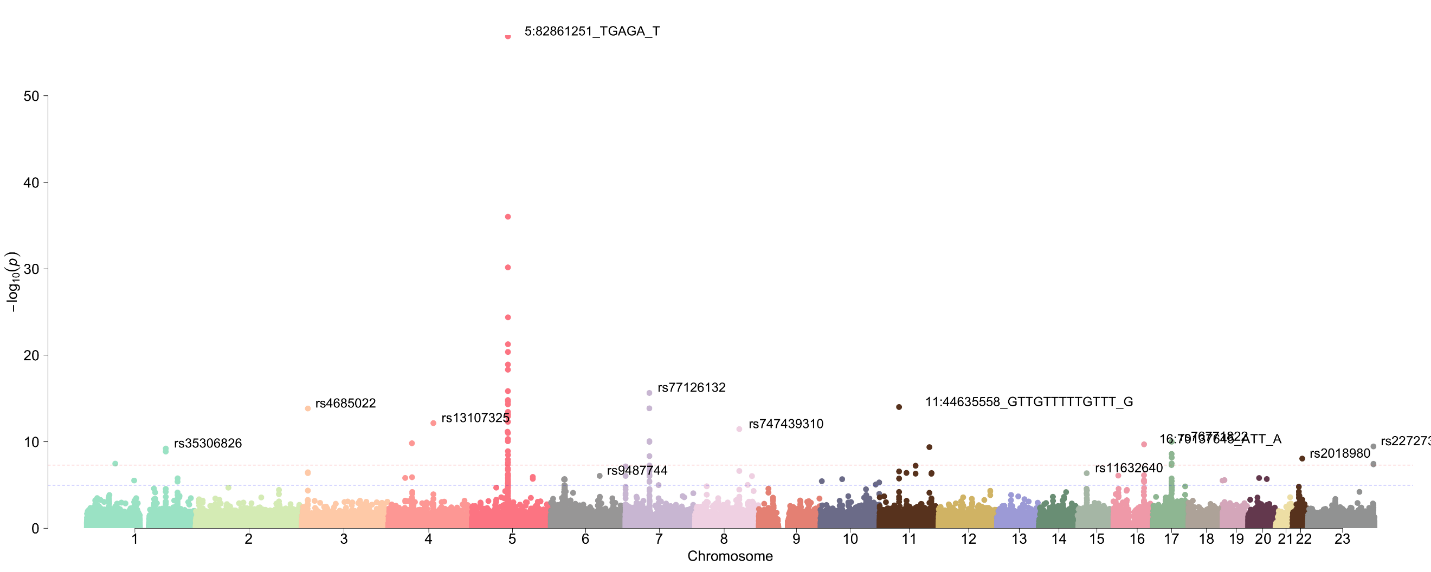


**Figure S8:** Manhattan plot of independent component 1 derived from the 33k sample. The red-dashed line indicates the Bonferroni corrected significance threshold (1.00e-7). The blue-dashed line indicates the suggestive significance threshold (1.00e-5). Note that our genome-wide data was heavily clumped at r^2^ < 0.1, which is why there are fewer SNPs with similar (low) p-values within each locus than in standard Manhattan plots.


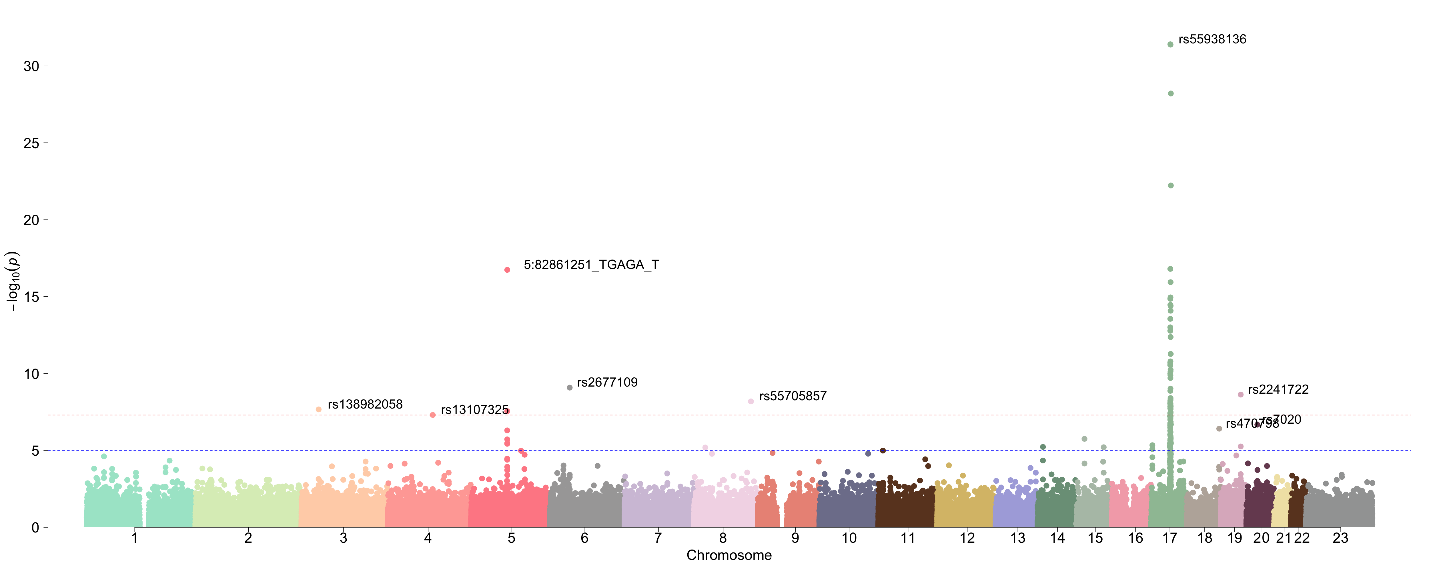


**Figure S9:** Manhattan plot of independent component 2 derived from the 33k sample. The red-dashed line indicates the Bonferroni corrected significance threshold (1.00e-7). The blue-dashed line indicates the suggestive significance threshold (1.00e-5). Note that our genome-wide data was heavily clumped at r^2^ < 0.1, which is why there are fewer SNPs with similar (low) p-values within each locus than in standard Manhattan plots.


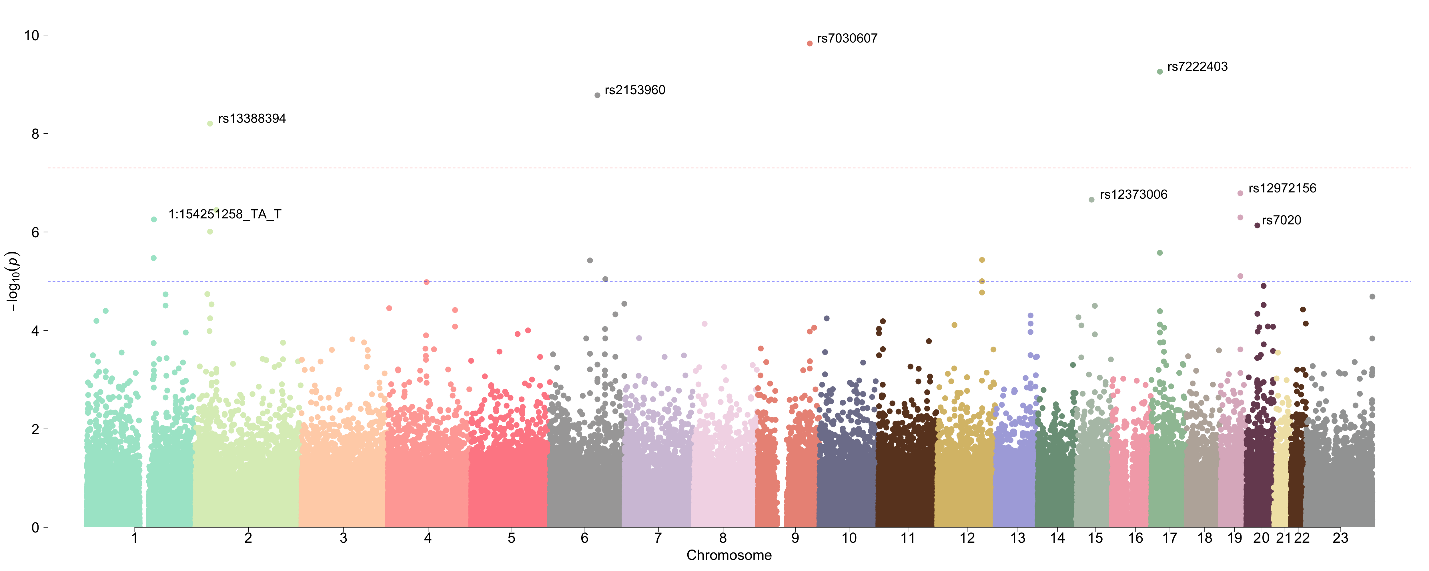


**Figure S10:** Manhattan plot of independent component 3 derived from the 33k sample. The red-dashed line indicates the Bonferroni corrected significance threshold (1.00e-7). The blue-dashed line indicates the suggestive significance threshold (1.00e-5). Note that our genome-wide data was heavily clumped at r^2^ < 0.1, which is why there are fewer SNPs with similar (low) p-values within each locus than in standard Manhattan plots.


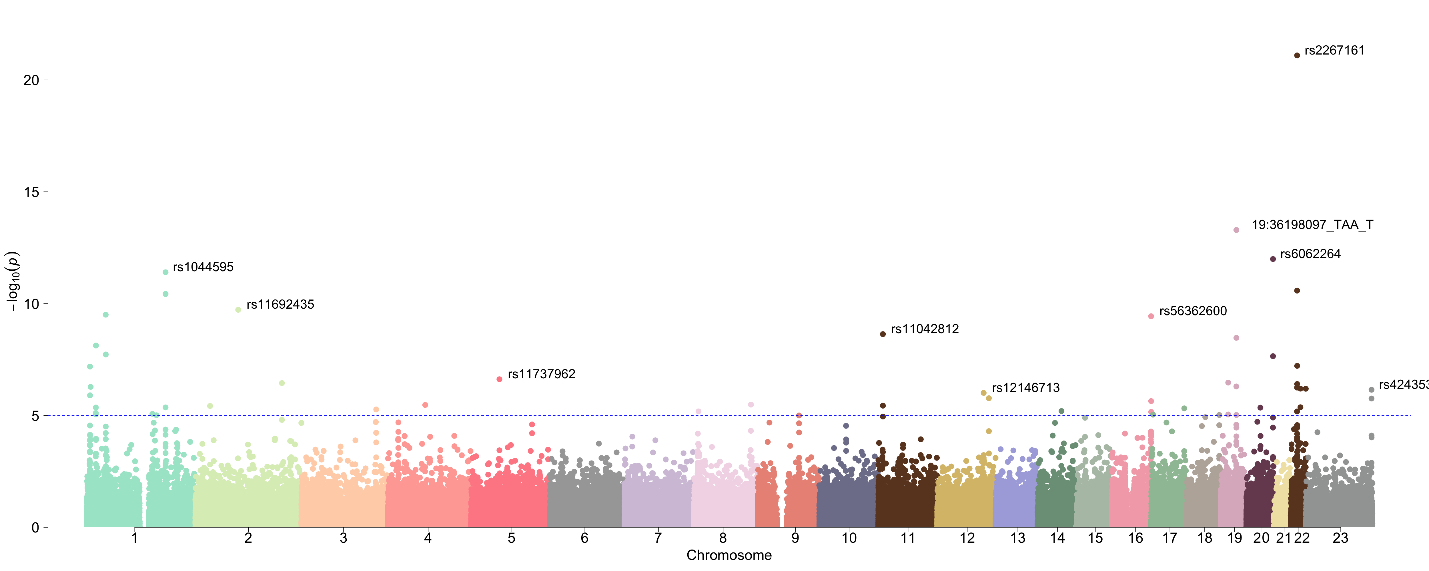


**Figure S11:** Manhattan plot of independent component 4 derived from the 33k sample. The red-dashed line indicates the Bonferroni corrected significance threshold (1.00e-7). The blue-dashed line indicates the suggestive significance threshold (1.00e-5). Note that our genome-wide data was heavily clumped at r^2^ < 0.1, which is why there are fewer SNPs with similar (low) p-values within each locus than in standard Manhattan plots.


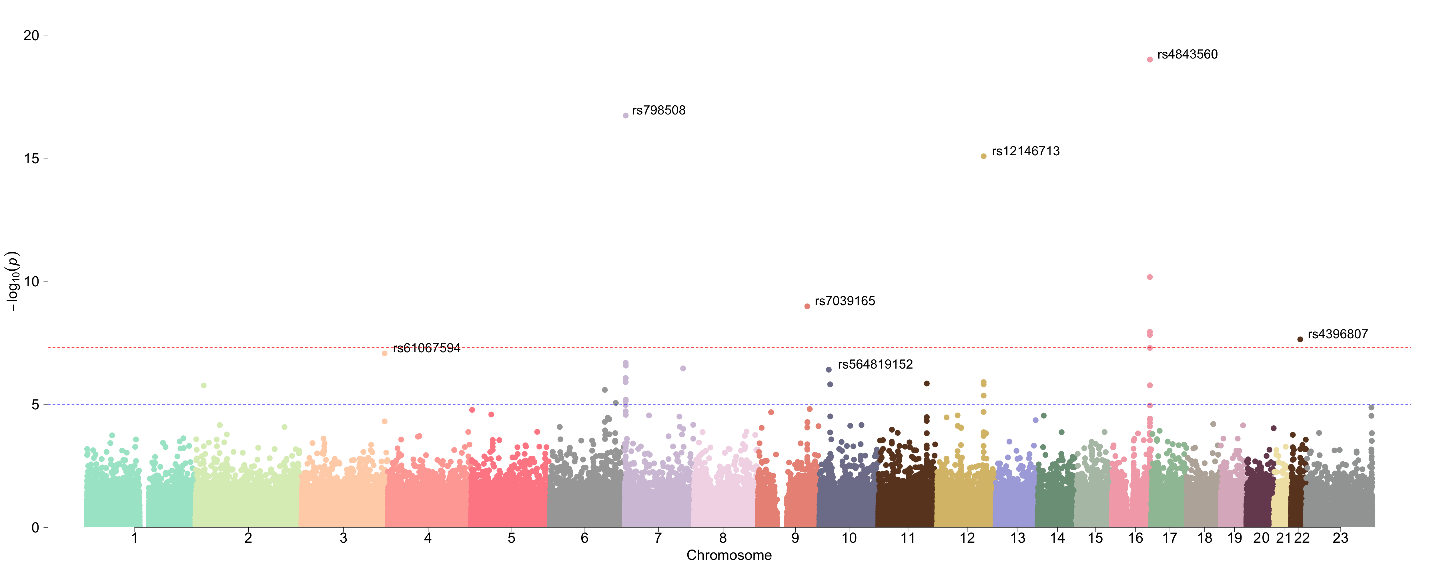


**Figure S12:** Manhattan plot of independent component 5 derived from the 33k sample. The red-dashed line indicates the Bonferroni corrected significance threshold (1.00e-7). The blue-dashed line indicates the suggestive significance threshold (1.00e-5). Note that our genome-wide data was heavily clumped at r^2^ < 0.1, which is why there are fewer SNPs with similar (low) p-values within each locus than in standard Manhattan plots.


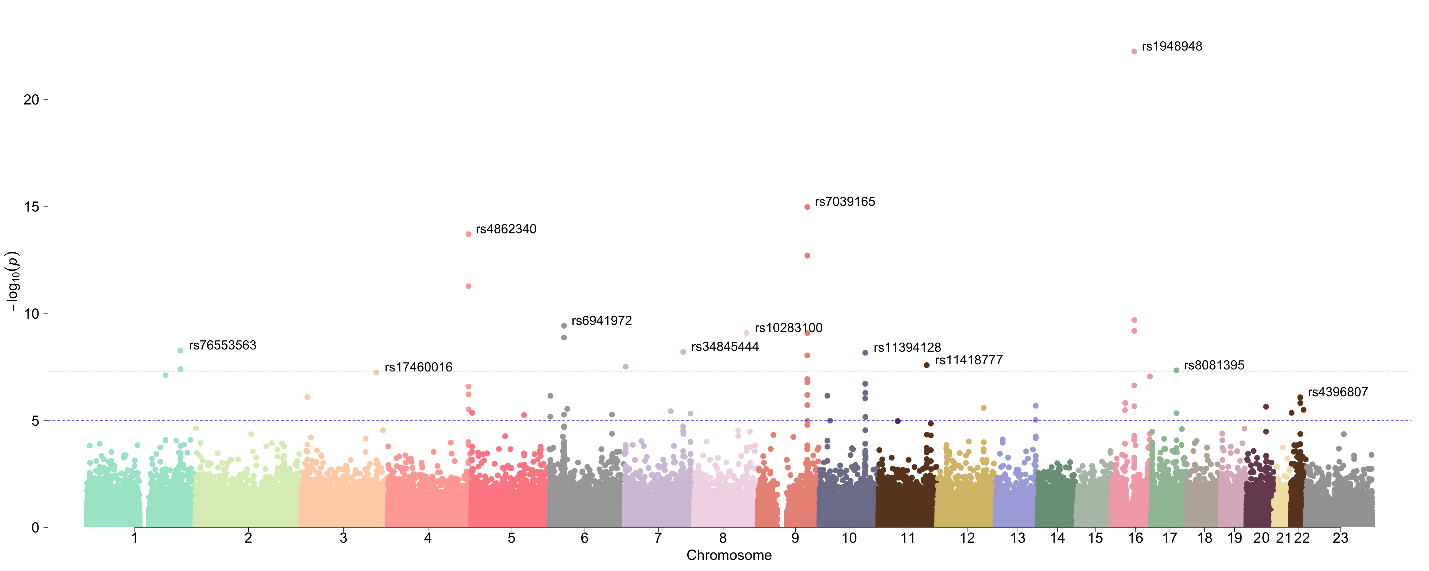


**Figure S13:** Manhattan plot of independent component 6 derived from the 33k sample. The red-dashed line indicates the Bonferroni corrected significance threshold (1.00e-7). The blue-dashed line indicates the suggestive significance threshold (1.00e-5). Note that our genome-wide data was heavily clumped at r^2^ < 0.1, which is why there are fewer SNPs with similar (low) p-values within each locus than in standard Manhattan plots.


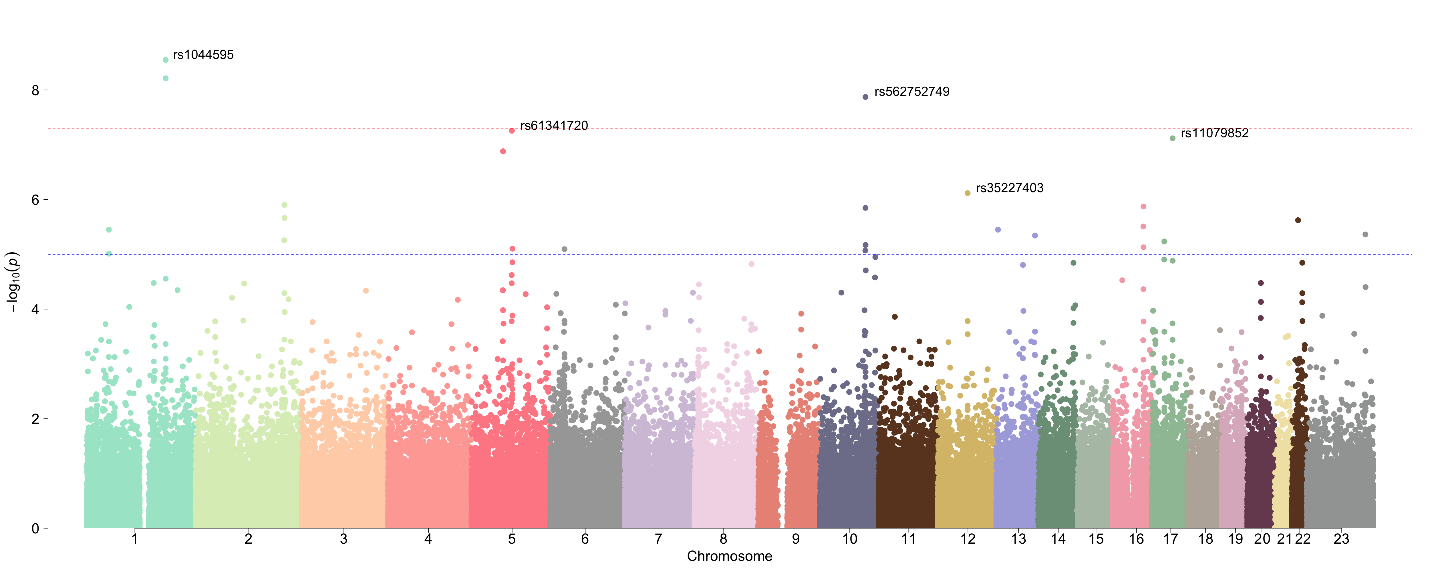


**Figure S14:** Manhattan plot of independent component 7 derived from the 33k sample. The red-dashed line indicates the Bonferroni corrected significance threshold (1.00e-7). The blue-dashed line indicates the suggestive significance threshold (1.00e-5). Note that our genome-wide data was heavily clumped at r^2^ < 0.1, which is why there are fewer SNPs with similar (low) p-values within each locus than in standard Manhattan plots.


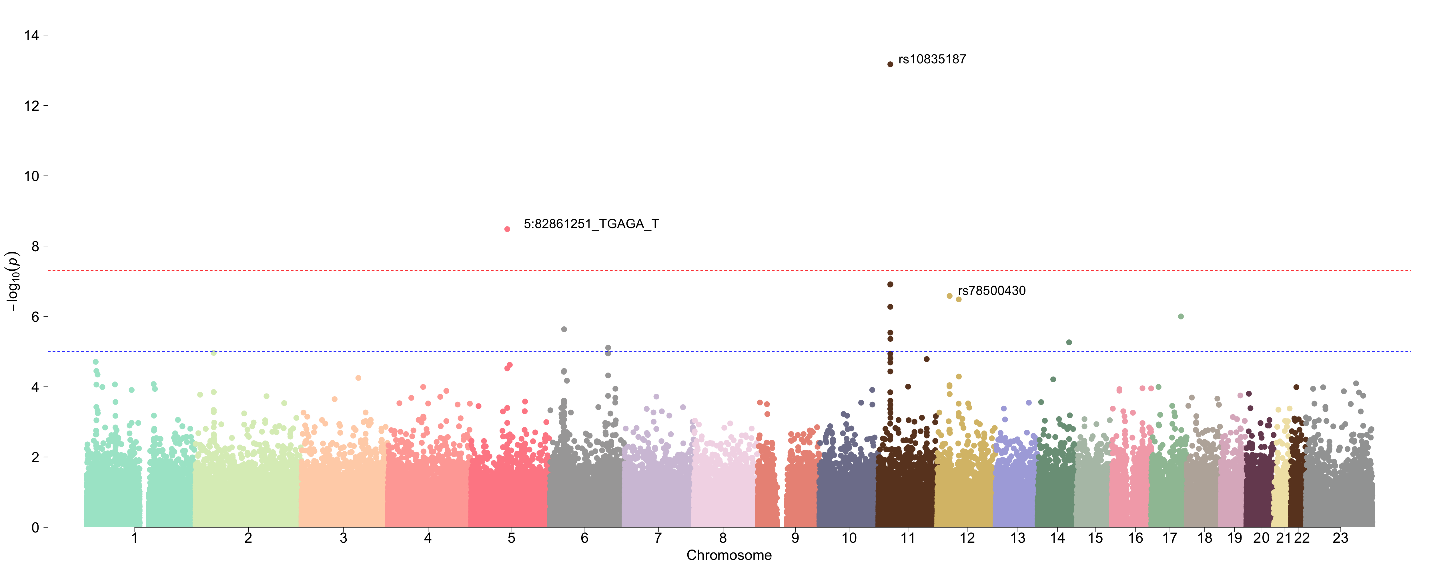


**Figure S15:** Manhattan plot of independent component 8 derived from the 33k sample. The red-dashed line indicates the Bonferroni corrected significance threshold (1.00e-7). The blue-dashed line indicates the suggestive significance threshold (1.00e-5). Note that our genome-wide data was heavily clumped at r^2^ < 0.1, which is why there are fewer SNPs with similar (low) p-values within each locus than in standard Manhattan plots.


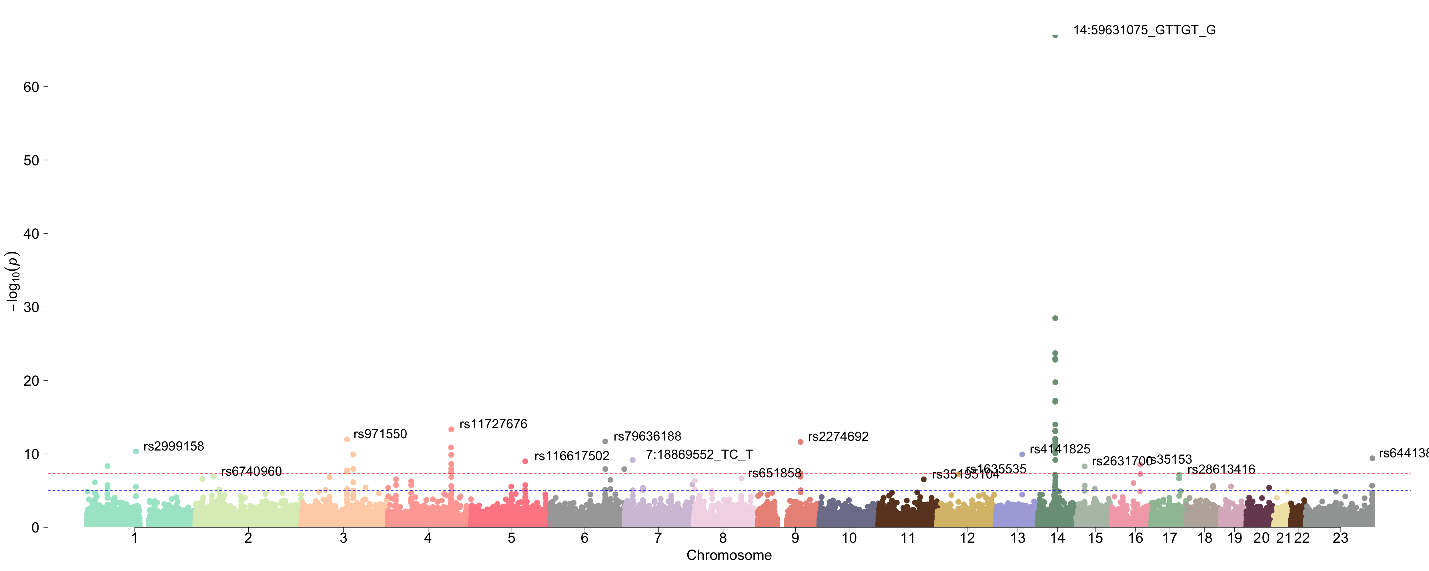


**Figure S16:** Manhattan plot of independent component 9 derived from the 33k sample. The red-dashed line indicates the Bonferroni corrected significance threshold (1.00e-7). The blue-dashed line indicates the suggestive significance threshold (1.00e-5). Note that our genome-wide data was heavily clumped at r^2^ < 0.1, which is why there are fewer SNPs with similar (low) p-values within each locus than in standard Manhattan plots.


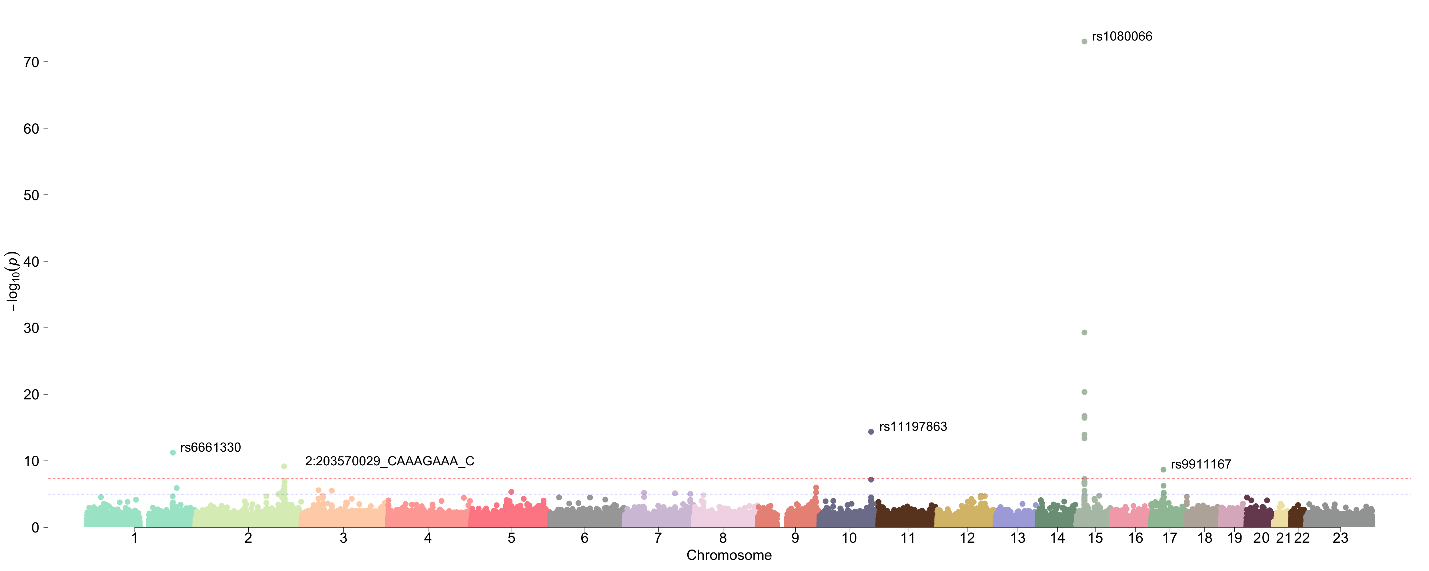


**Figure S17:** Manhattan plot of independent component 10 derived from the 33k sample. The red-dashed line indicates the Bonferroni corrected significance threshold (1.00e-7). The blue-dashed line indicates the suggestive significance threshold (1.00e-5). Note that our genome-wide data was heavily clumped at r^2^ < 0.1, which is why there are fewer SNPs with similar (low) p-values within each locus than in standard Manhattan plots.

**PCA Manhattan plots**


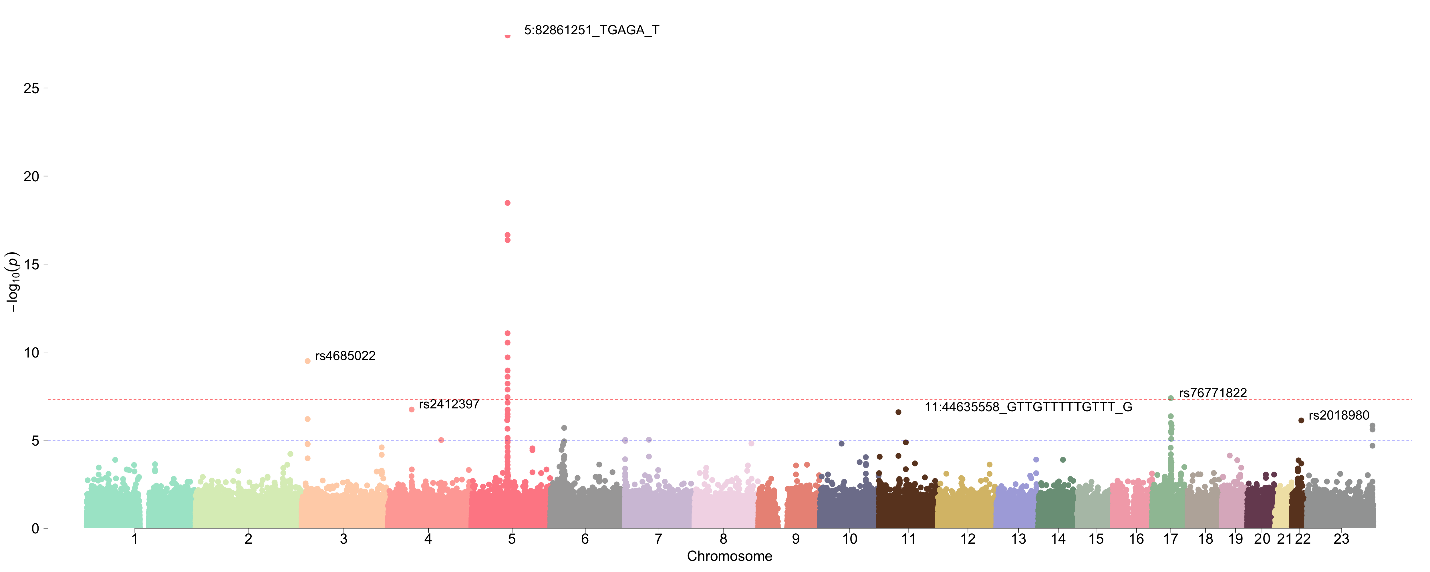


**Figure S18:** Manhattan plot of principal component 1 derived from the 33k sample. The red-dashed line indicates the Bonferroni corrected significance threshold (1.00e-7). The blue-dashed line indicates the suggestive significance threshold (1.00e-5). Note that our genome-wide data was heavily clumped at r^2^ < 0.1, which is why there are fewer SNPs with similar (low) p-values within each locus than in standard Manhattan plots.


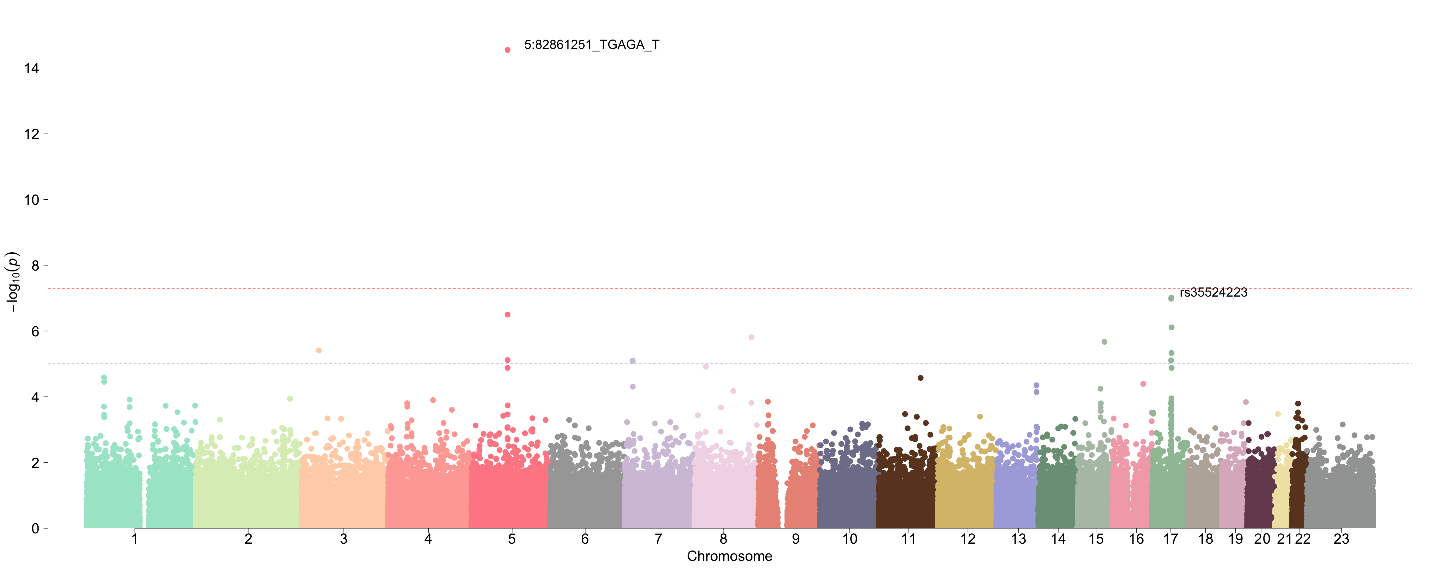


**Figure S19:** Manhattan plot of principal component 2 derived from the 33k sample. The red-dashed line indicates the Bonferroni corrected significance threshold (1.00e-7). The blue-dashed line indicates the suggestive significance threshold (1.00e-5). Note that our genome-wide data was heavily clumped at r^2^ < 0.1, which is why there are fewer SNPs with similar (low) p-values within each locus than in standard Manhattan plots.


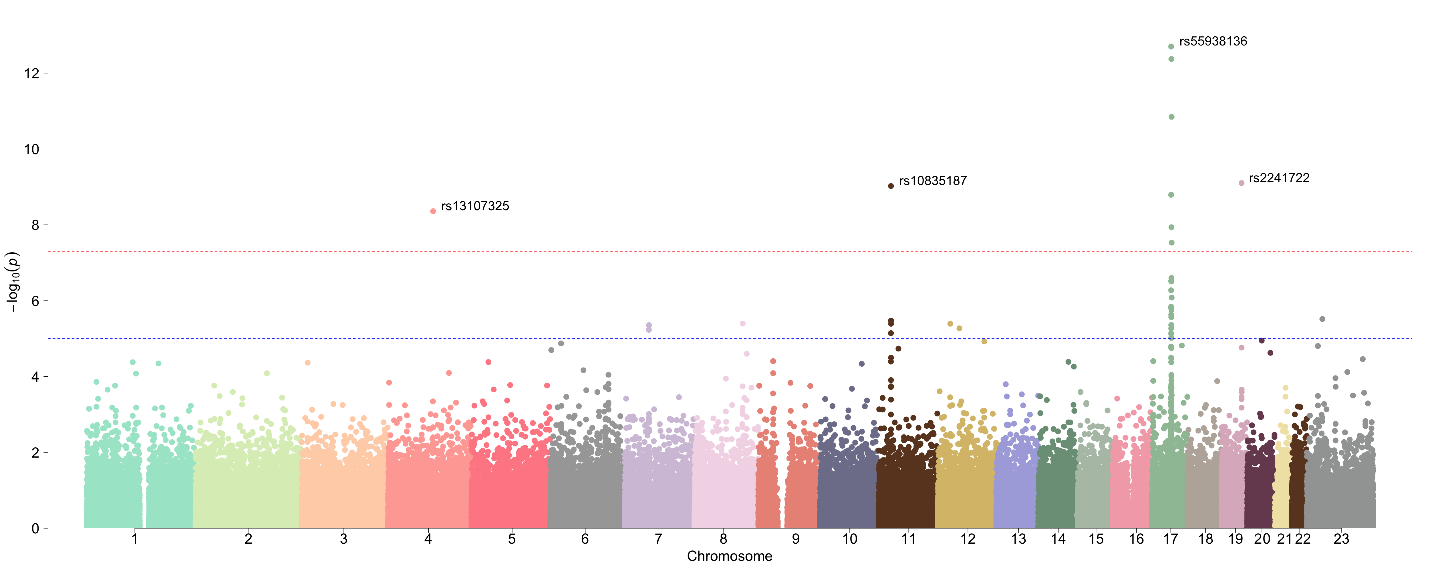


**Figure S20:** Manhattan plot of principal component 3 derived from the 33k sample. The red-dashed line indicates the Bonferroni corrected significance threshold (1.00e-7). The blue-dashed line indicates the suggestive significance threshold (1.00e-5). Note that our genome-wide data was heavily clumped at r^2^ < 0.1, which is why there are fewer SNPs with similar (low) p-values within each locus than in standard Manhattan plots.


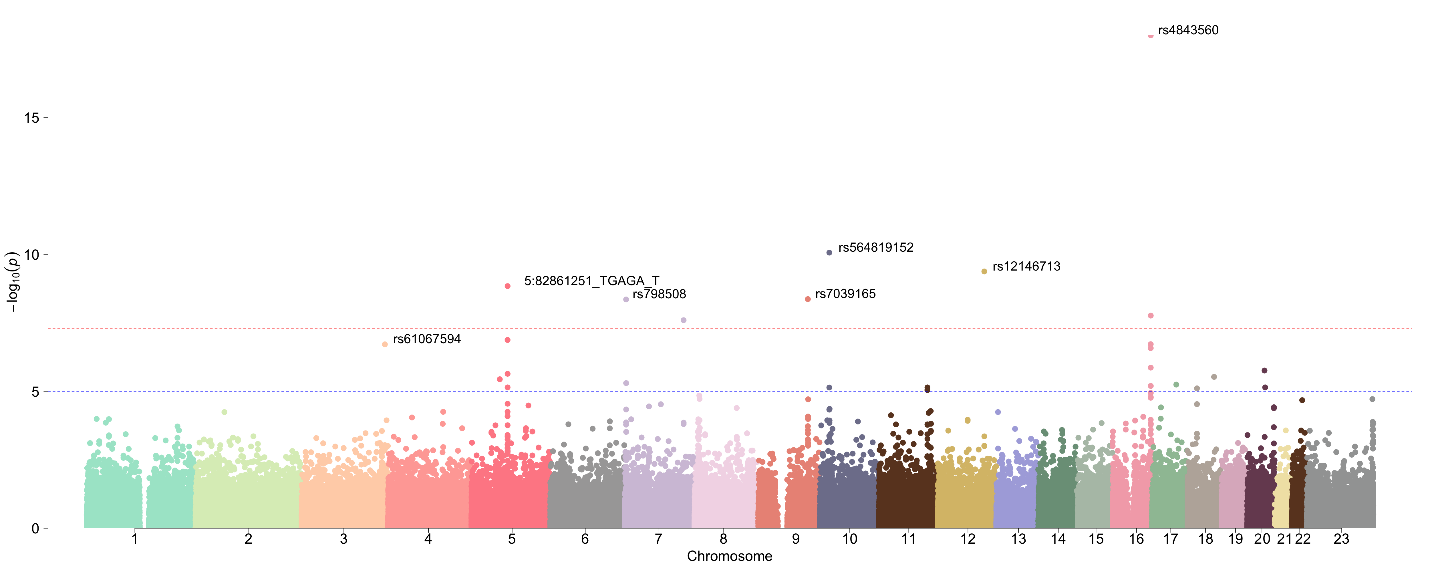


**Figure S21:** Manhattan plot of principal component 4 derived from the 33k sample. The red-dashed line indicates the Bonferroni corrected significance threshold (1.00e-7). The blue-dashed line indicates the suggestive significance threshold (1.00e-5). Note that our genome-wide data was heavily clumped at r^2^ < 0.1, which is why there are fewer SNPs with similar (low) p-values within each locus than in standard Manhattan plots.


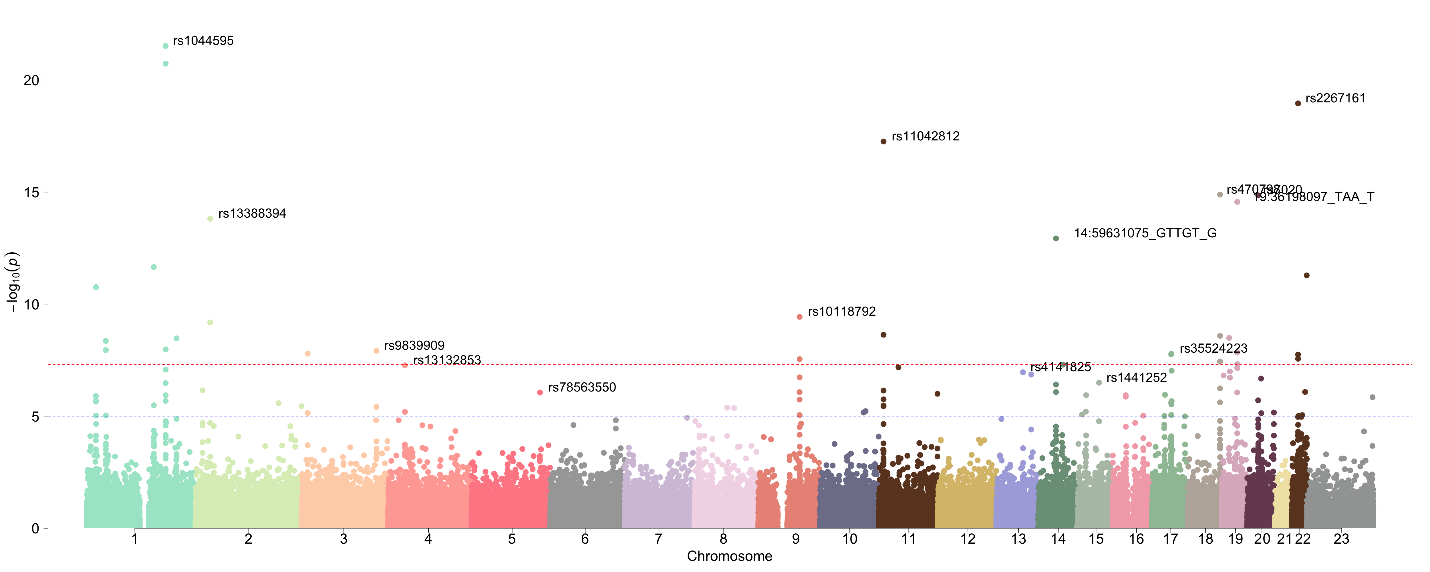


**Figure S22:** Manhattan plot of principal component 5 derived from the 33k sample. The red-dashed line indicates the Bonferroni corrected significance threshold (1.00e-7). The blue-dashed line indicates the suggestive significance threshold (1.00e-5). Note that our genome-wide data was heavily clumped at r^2^ < 0.1, which is why there are fewer SNPs with similar (low) p-values within each locus than in standard Manhattan plots.


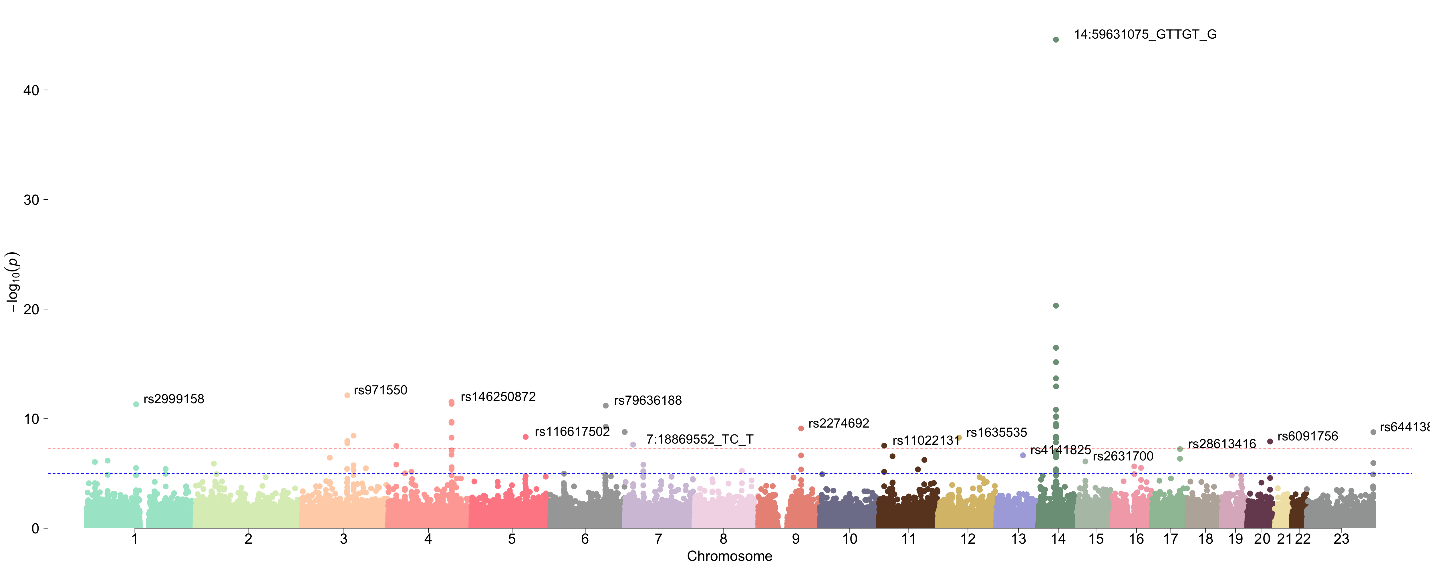


**Figure S23:** Manhattan plot of principal component 6 derived from the 33k sample. The red-dashed line indicates the Bonferroni corrected significance threshold (1.00e-7). The blue-dashed line indicates the suggestive significance threshold (1.00e-5). Note that our genome-wide data was heavily clumped at r^2^ < 0.1, which is why there are fewer SNPs with similar (low) p-values within each locus than in standard Manhattan plots.


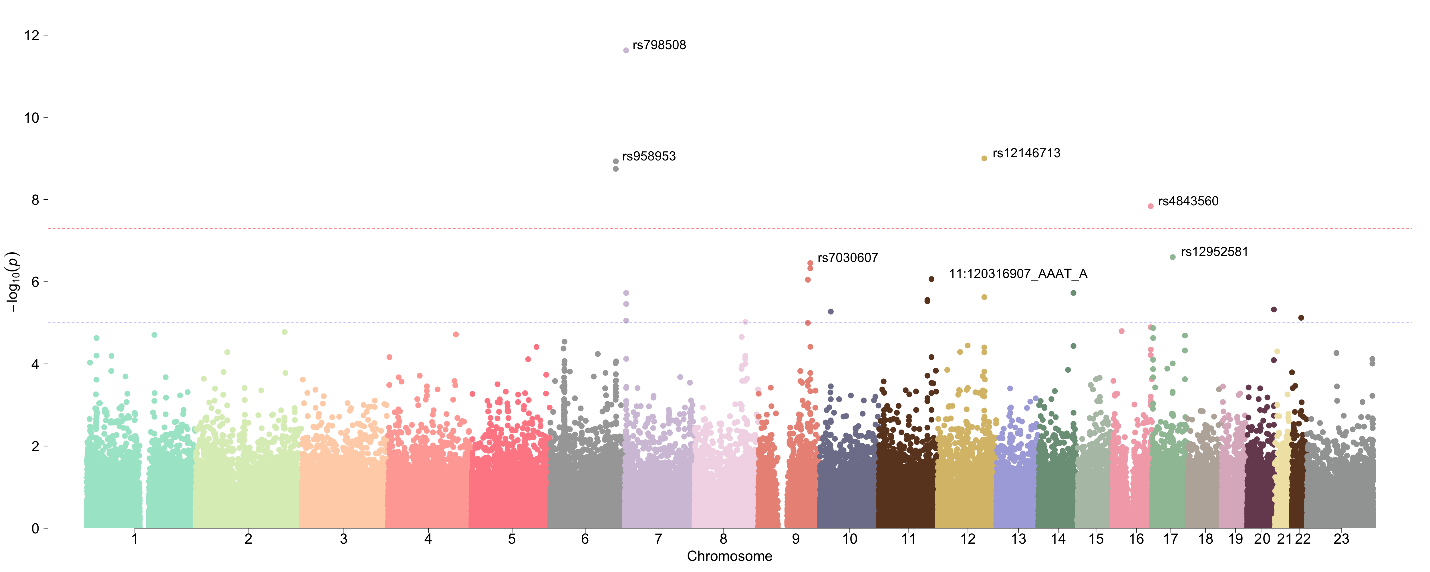


**Figure S24:** Manhattan plot of principal component 7 derived from the 33k sample. The red-dashed line indicates the Bonferroni corrected significance threshold (1.00e-7). The blue-dashed line indicates the suggestive significance threshold (1.00e-5). Note that our genome-wide data was heavily clumped at r^2^ < 0.1, which is why there are fewer SNPs with similar (low) p-values within each locus than in standard Manhattan plots.


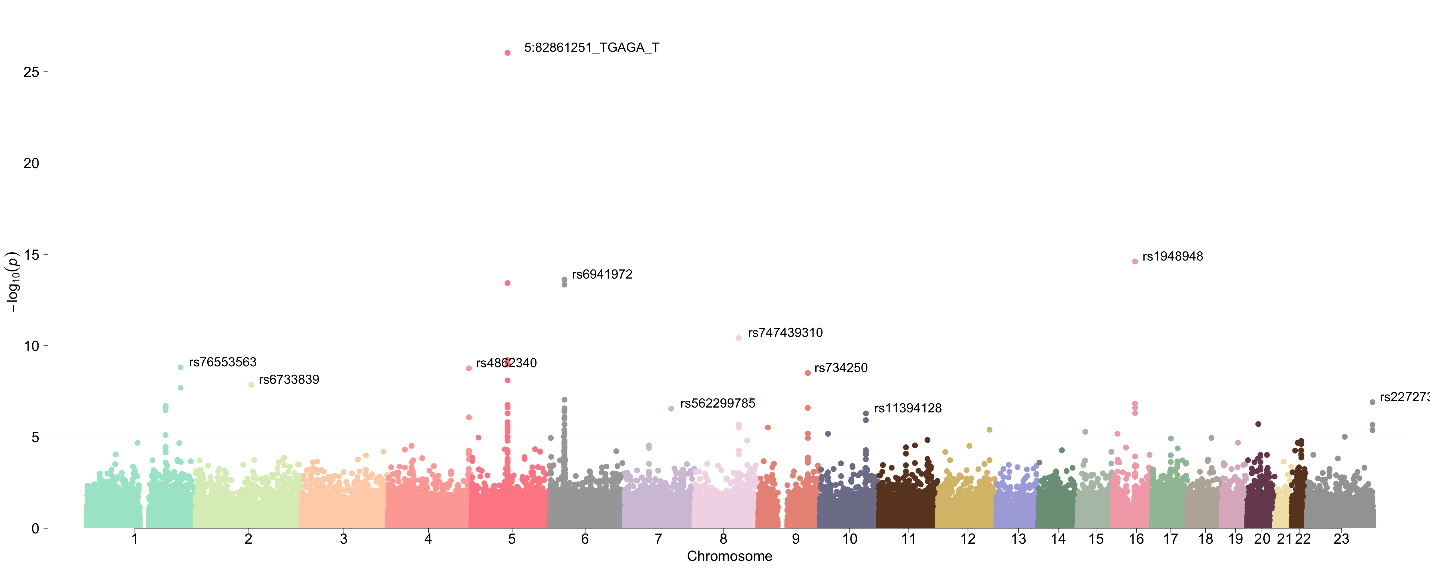


**Figure S25:** Manhattan plot of principal component 8 derived from the 33k sample. The red-dashed line indicates the Bonferroni corrected significance threshold (1.00e-7). The blue-dashed line indicates the suggestive significance threshold (1.00e-5). Note that our genome-wide data was heavily clumped at r^2^ < 0.1, which is why there are fewer SNPs with similar (low) p-values within each locus than in standard Manhattan plots.


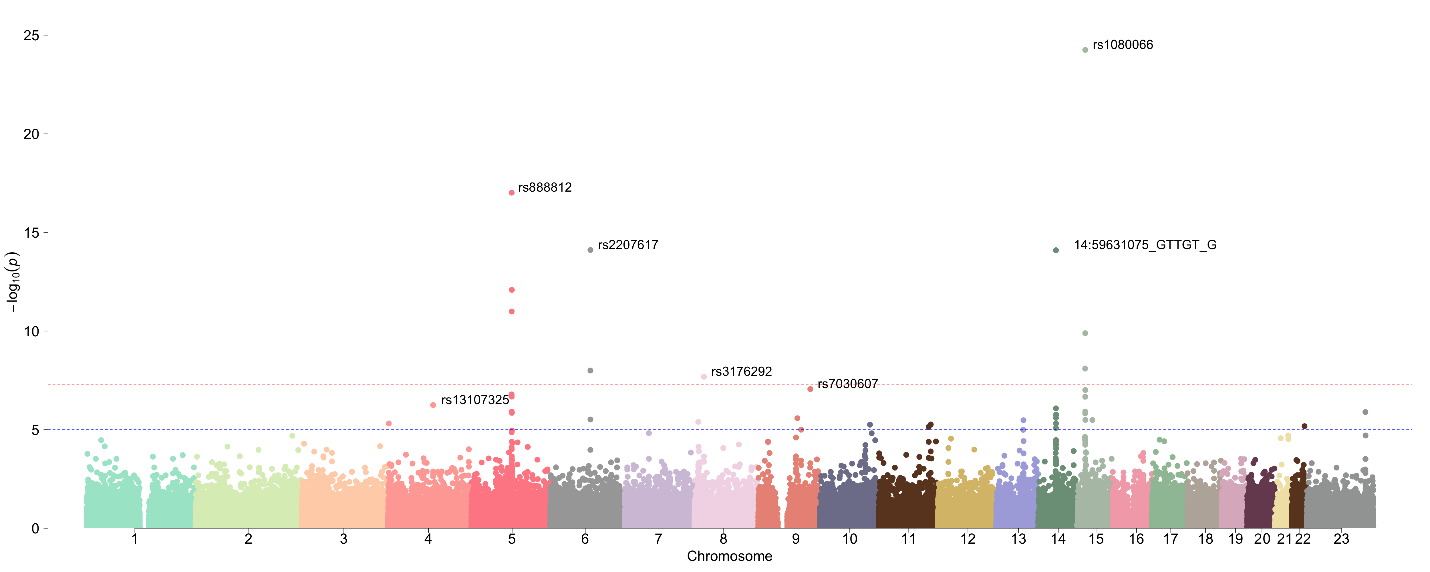


**Figure S26:** Manhattan plot of principal component 9 derived from the 33k sample. The red-dashed line indicates the Bonferroni corrected significance threshold (1.00e-7). The blue-dashed line indicates the suggestive significance threshold (1.00e-5). Note that our genome-wide data was heavily clumped at r^2^ < 0.1, which is why there are fewer SNPs with similar (low) p-values within each locus than in standard Manhattan plots.


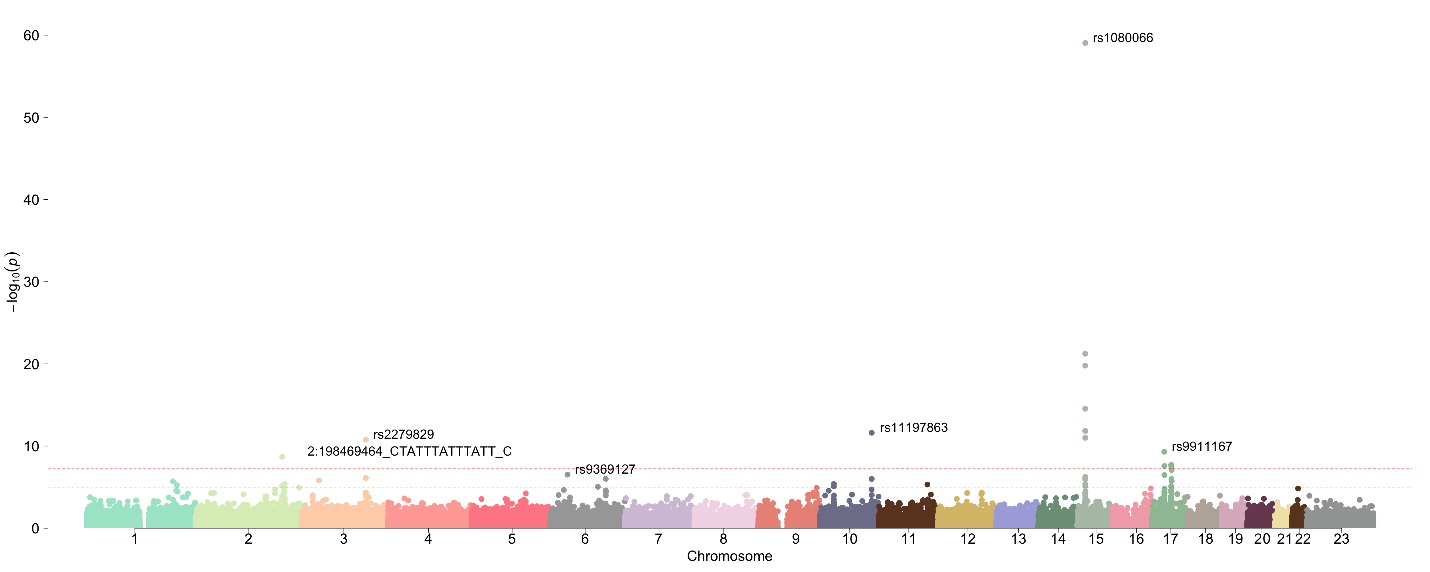


**Figure S27:** Manhattan plot of principal component 10 derived from the 33k sample. The red-dashed line indicates the Bonferroni corrected significance threshold (1.00e-7). The blue-dashed line indicates the suggestive significance threshold (1.00e-5). Note that our genome-wide data was heavily clumped at r^2^ < 0.1, which is why there are fewer SNPs with similar (low) p-values within each locus than in standard Manhattan plots.

**Tables**

**Table S1**: Statistics of Principal Component reproducibility at dimension 50. The 22k sample = discovery sample; 11k sample = replication sample. P-values are adjusted for MCC as specified in the methods section in the main manuscript. 20 strongest component correlations are shown. The kurtotic components of the raw decomposition made a Fisher’s test inappropriate.

| **Z-transformed Decomposition** | | | | | **Raw Decomposition** | | | |
| --- | --- | --- | --- | --- | --- | --- | --- | --- |
| **22k sample component** | **11k sample component** | **Coefficient (Pearson's \|r\|)** | ***Correlation p*-value** | **Fisher's *p*-value** | **22k sample component** | **11k sample component** | **Coefficient (Pearson's \|r\|)** | ***Correlation p*-value** |
| PC1 | PC1 | 0.33 | <10^-308^ | 8.16^-246^ | PC1 | PC1 | 0.30 | <10^-308^ |
| PC2 | PC2 | 0.25 | <10^-308^ | 6.69^-47^ | PC2 | PC2 | 0.23 | <10^-308^ |
| PC3 | PC3 | 0.26 | <10^-308^ | 1.15^-38^ | PC3 | PC3 | 0.24 | <10^-308^ |
| PC4 | PC4 | 0.21 | <10^-308^ | 1.19^-35^ | PC4 | PC4 | 0.17 | <10^-308^ |
| PC5 | PC5 | 0.18 | <10^-308^ | 1.22^-28^ | PC5 | PC5 | 0.13 | <10^-308^ |
| PC6 | PC6 | 0.16 | <10^-308^ | 2.95^-19^ | PC6 | PC6 | 0.11 | <10^-308^ |
| PC7 | PC7 | 0.17 | <10^-308^ | 5.30^-19^ | PC7 | PC7 | 0.13 | <10^-308^ |
| PC8 | PC8 | 0.15 | <10^-308^ | 1.31^-23^ | PC8 | PC8 | 0.10 | <10^-308^ |
| PC9 | PC9 | 0.13 | <10^-308^ | 0.03 | PC9 | PC9 | 0.08 | 2.17^-256^ |
| PC10 | PC10 | 0.12 | <10^-308^ | 5.14^-08^ | PC14 | PC14 | 0.08 | 3.22^-220^ |
| PC13 | PC13 | 0.11 | <10^-308^ | 0.003 | PC12 | PC12 | 0.08 | 1.84^-202^ |
| PC14 | PC14 | 0.12 | <10^-308^ | 0.0001 | PC13 | PC13 | 0.07 | 1.69^-199^ |
| PC15 | PC15 | 0.11 | <10^-308^ | 0.0077 | PC10 | PC10 | 0.07 | 1.13^-196^ |
| PC20 | PC20 | 0.09 | 8.22^-306^ | >0.05 | PC15 | PC15 | 0.07 | 7.60^-194^ |
| PC19 | PC19 | 0.09 | 3.79^-288^ | >0.05 | PC11 | PC11 | 0.06 | 1.80^-135^ |
| PC18 | PC18 | 0.09 | 7.33^-267^ | >0.05 | PC17 | PC16 | 0.05 | 6.99^-107^ |
| PC16 | PC17 | 0.08 | 7.30^-260^ | >0.05 | PC19 | PC19 | 0.05 | 2.51^-100^ |
| PC17 | PC16 | 0.08 | 1.25^-243^ | >0.05 | PC22 | PC22 | 0.05 | 3.25^-90^ |
| PC24 | PC23 | 0.07 | 2.80^-177^ | >0.05 | PC20 | PC20 | 0.05 | 1.56^-87^ |

**Table S2**: Statistics of Independent Component reproducibility at dimension 50. The 22k sample = discovery sample; 11k sample = replication sample. P-values are adjusted for MCC as specified in the methods section in the main manuscript. 20 strongest component correlations are shown. The kurtotic components of the raw decomposition made a Fisher’s test inappropriate.

| **Z-transformed Decomposition** | | | | | **Raw Decomposition** | | | |
| --- | --- | --- | --- | --- | --- | --- | --- | --- |
| **22k sample component** | **11k sample component** | **Coefficient (Pearson's \|r\|)** | ***Correlation p*-value** | **Fisher's *p*-value** | **22k sample component** | **11k sample component** | **Coefficient (Pearson's \|r\|)** | ***Correlation p*-value** |
| IC1 | IC2 | 0.23 | <10^-308^ | 5.47^-66^ | IC1 | IC1 | 0.19 | <10^-308^ |
| IC6 | IC6 | 0.20 | <10^-308^ | 1.01^-11^ | IC2 | IC1 | 0.09 | <10^-308^ |
| IC9 | IC9 | 0.15 | <10^-308^ | 7.30^-07^ | IC7 | IC2 | 0.13 | <10^-308^ |
| IC7 | IC7 | 0.14 | <10^-308^ | >0.05 | IC2 | IC3 | 0.10 | <10^-308^ |
| IC10 | IC10 | 0.12 | <10^-308^ | 0.003 | IC4 | IC3 | 0.12 | <10^-308^ |
| IC2 | IC5 | 0.11 | <10^-308^ | >0.05 | IC4 | IC4 | 0.10 | <10^-308^ |
| IC5 | IC7 | 0.10 | <10^-308^ | >0.05 | IC3 | IC5 | 0.16 | <10^-308^ |
| IC2 | IC3 | 0.11 | <10^-308^ | >0.05 | IC8 | IC6 | 0.10 | <10^-308^ |
| IC8 | IC3 | 0.12 | <10^-308^ | >0.05 | IC6 | IC7 | 0.19 | <10^-308^ |
| IC5 | IC5 | 0.13 | <10^-308^ | 0.005 | IC5 | IC8 | 0.09 | 7.98^-305^ |
| IC3 | IC1 | 0.17 | <10^-308^ | >0.05 | IC2 | IC4 | 0.09 | 1.72^-293^ |
| IC4 | IC4 | 0.17 | <10^-308^ | >0.05 | IC4 | IC9 | 0.08 | 4.48^-203^ |
| IC8 | IC8 | 0.18 | <10^-308^ | >0.05 | IC2 | IC2 | 0.07 | 2.44^-183^ |
| IC7 | IC5 | 0.09 | 3.75^-296^ | >0.05 | IC4 | IC10 | 0.07 | 9.11^-174^ |
| IC2 | IC4 | 0.09 | 1.58^-276^ | >0.05 | IC9 | IC9 | 0.07 | 1.34^-171^ |
| IC4 | IC3 | 0.09 | 2.20^-275^ | >0.05 | IC8 | IC4 | 0.07 | 1.48^-164^ |
| IC1 | IC5 | 0.08 | 2.10^-257^ | >0.05 | IC1 | IC3 | 0.07 | 6.76^-160^ |
| IC4 | IC6 | 0.08 | 5.23^-226^ | >0.05 | IC9 | IC7 | 0.07 | 7.95^-158^ |
| IC2 | IC8 | 0.07 | 3.05^-196^ | >0.05 | IC5 | IC6 | 0.07 | 1.88^-157^ |

**Table S3**: Table containing the correlation coefficients and p-values between the genomic independent component IDP-loadings and the reproducibility of IDP-specific genetic effects between the univariate GWASs of the 11k and 22k samples. The Index column indicates which component was correlated with the univariate GWAS reproducibility.

| Correlation Coefficient | P-Value | Index |
| --- | --- | --- |
| 0.637528374 | 1.10E-255 | IC1 vs UnivarGWAS_reproc |
| 0.40001543 | 8.38E-87 | IC2 vs UnivarGWAS_reproc |
| 0.298918799 | 1.92E-47 | IC3 vs UnivarGWAS_reproc |
| 0.632017165 | 5.45E-250 | IC4 vs UnivarGWAS_reproc |
| 0.204053216 | 1.79E-22 | IC5 vs UnivarGWAS_reproc |
| 0.383628167 | 2.05E-79 | IC6 vs UnivarGWAS_reproc |
| 0.204031501 | 1.81E-22 | IC7 vs UnivarGWAS_reproc |
| 0.173633967 | 1.29E-16 | IC8 vs UnivarGWAS_reproc |
| -0.090617621 | 1.75E-05 | IC9 vs UnivarGWAS_reproc |
| 0.113250261 | 7.74E-08 | IC10 vs UnivarGWAS_reproc |

**Table S4**: Table containing the correlation coefficients and p-values between the genomic principal component IDP-loadings and the reproducibility of IDP-specific genetic effects between the univariate GWASs of the 11k and 22k samples. The Index column indicates which component was correlated with the univariate GWAS reproducibility.

| Correlation Coefficient | P-Value | Index |
| --- | --- | --- |
| 0.702481091 | <1.00E-308 | PC1 vs UnivarGWAS_reproc |
| 0.429346859 | 4.14E-101 | PC2 vs UnivarGWAS_reproc |
| -0.112386945 | 9.72E-08 | PC3 vs UnivarGWAS_reproc |
| 0.046431321 | 0.028020544 | PC4 vs UnivarGWAS_reproc |
| 0.127797263 | 1.29E-09 | PC5 vs UnivarGWAS_reproc |
| -0.041431823 | 0.049969976 | PC6 vs UnivarGWAS_reproc |
| 0.017625377 | 0.404507609 | PC7 vs UnivarGWAS_reproc |
| 0.092884622 | 1.07E-05 | PC8 vs UnivarGWAS_reproc |
| -0.078763427 | 0.000190987 | PC9 vs UnivarGWAS_reproc |
| -0.056281166 | 0.007727713 | PC10 vs UnivarGWAS_reproc |

**Computing Resources**

The genomic PCA/ICA algorithm is memory hungry, and should be run on an appropriate, high performance computing environment. Genomic PCA/ICA uses the MELODIC algorithm of FSL (https://fsl.fmrib.ox.ac.uk/fsl/fslwiki/MELODIC). Increasing numbers of reconstructed components (i.e. dimensionality) and input data size will increase the memory requirements. In the present analysis we first applied strict clumping thresholds to remove redundant information due to LD and reduce the SNP-dimension. Next, we decomposed the 4 dimensional array of 2240 GWAS summary statistics across 165,364 SNPs. This array is ~143 GB in size and formatted as a g-zipped NifTI file. For decompositions in the upper bounds applied in this analysis, specifically where the data is decomposed into 200 PCs and ICs, we suggest the following: The task should be submitted to a large computing node with several cores suitable for parallelization. One run requires ~600 GB of RAM-memory, with a runtime of ~5 hours. Testing the variance explained against model complexity to determine the most promising dimensionality for the present data requires ~10 runs.

**References**

Galwey, N. W. (2009). A new measure of the effective number of tests, a practical tool for comparing families of non-independent significance tests. *Genetic Epidemiology*, *33*(7), 559–568. https://doi.org/10.1002/gepi.20408

Gao, X., Starmer, J., & Martin, E. R. (2008). A multiple testing correction method for genetic association studies using correlated single nucleotide polymorphisms. *Genetic Epidemiology*, *32*(4), 361–369. https://doi.org/10.1002/gepi.20310
